# HTR2B-Mediated Endothelial Protection: Modulating Ferroptosis via the PI3K/AKT Signaling Pathway in Atherosclerosis

**DOI:** 10.1155/cdr/7934590

**Published:** 2025-10-03

**Authors:** Delin Li, Hui Li, Quanwei Zhao, Caiwei Gong, Long Chen, Chengzhu Xiong, Shaoliang Shen, Fujun Liao, Wupeng Liu, Danan Liu

**Affiliations:** ^1^Department of Cardiovascularology, The Affiliated Hospital of Guizhou Medical University, Guiyang, Guizhou, China; ^2^The Science and Technology Institute of Guizhou Medical University, Guiyang, Guizhou, China; ^3^Department of Cardiovascularology, Guihang Guiyang Hospital, Guiyang, Guizhou, China

**Keywords:** atherosclerosis, ferroptosis, HTR2B, low-density lipoprotein (LDL), PI3K/AKT

## Abstract

**Objective:**

The current study is aimed at elucidating the mechanisms underlying the involvement of ferroptosis in atherosclerosis (AS) and exploring potential therapeutic targets.

**Methods:**

Endothelial function and lipid peroxidation were assessed in vitro using HCAECs treated with OX-LDL. SLC7A11, GPX4, TfR1, and FTH1 were analyzed by western blot, respectively. Representative markers of ferroptosis including LDH, MDA, 4-HNE, GSH, and iron content were detected. HTR2B miRNA (OE-HTR2B) and controls (OE-NC, empty vector) were transfected. AS was induced in ApoE^−/−^ mice through a high-fat diet. The effect of ferroptosis inhibition on atherosclerotic lesion development was evaluated by different inhibitor treatments.

**Results:**

RNA-Seq analysis revealed dysregulated *HTR2B* expression in HCAECs exposed to OX-LDL, indicating its involvement in AS pathogenesis. OX-LDL exposure reduced cell viability and induced ferroptosis, characterized by decreased SLC7A11 and GPX4 expression and increased lipid peroxidation. Overexpression of *HTR2B* rescued cell viability, reduced Fe^2+^ accumulation, and upregulated SLC7A11 and GPX4, suggesting a protective role against ferroptosis. Further, *HTR2B* regulated ferroptosis via the PI3K/AKT pathway, as evidenced by changes in pathway protein phosphorylation. By activating HTR2B with an agonist BW-723C86, we verified that *HTR2B* can inhibit ferroptosis through the PI3K/AKT pathway in an atherosclerotic mouse model.

**Conclusion:**

HTR2B suppressed ferroptosis by promoting the PI3K/AKT axis, enhanced cellular viability, and exhibited a protective role in AS.

## 1. Introduction

Atherosclerosis (AS) is a systemic chronic vascular inflammatory disease that mainly involves the intima and medial layers of the arterial wall and is a major cause of the pathogenesis of many cardiovascular and cerebrovascular diseases [1, 2]. The main features of AS are endothelial dysfunction, lipid accumulation, inflammatory cell infiltration, and vascular smooth muscle cell (VSMC) proliferation. Atherosclerotic plaque formation occurs in four stages: fatty streaks, atherosclerotic plaques, complex atherosclerotic plaques, and clinical complications. Endothelial cells (ECs), VSMCs, and monocytes/macrophages are involved in plaque formation; meanwhile, plaque rupture leads to acute occlusion of the artery and myocardial infarction [3]. The therapeutic approach toward AS has witnessed a giant improvement in recent decades. Statins, ACE inhibitors, ARBs, calcium channel blockers, diuretics, antiplatelet drugs, and interventional therapy have largely improved the prognosis for AS patients, whereas investigation into novel therapeutic approaches remains necessary [4, 5]. Notably, the continuous exploration into the mechanisms underlying AS is vital for the optimism of AS management.

Ferroptosis, which is a form of regulated cell death characterized by iron-dependent lipid peroxidation engaged in the pathogenesis of cancers and acute injury [6, 7], typically plays a key role in the development and progression of atherosclerotic lesions by affecting the stability of arterial plaque and the inflammatory environment within the vessel walls. Its enrollment in AS has been investigated: Bai et al. demonstrate that inhibiting iron death alleviates AS by attenuating lipid peroxidation and endothelial dysfunction in mouse aortic ECs [8]. Xiang et al. illustrate that SLC3A2 contributes to EC damage and acceleration of AS plaque development by promoting endothelial ferroptosis in vivo [9]. Given the dysregulation of ferroptosis in AS, there is growing interest in this pathway as a potential therapeutic strategy for AS. HTR2B is first recognized as a serotonin receptor, which serves multifarious functions in psychiatric disorders and pain modulation [10]. Accumulated evidence highlights its involvement in influencing mood, cognition, and potentially metabolic functions. More importantly, emerging evidence suggests a complex interplay between HTR2B, PI3K-AKT signaling, and ferroptosis, implicating their coregulation in neurological conditions and cancer progression: Jiang et al. demonstrate that under 5-HT stimulation, HTR2B enhances the PI3K/AKT axis in pancreatic cancer mice [11]. Similarly, Tu et al. elucidate that HTR2B collaborates with Fyn to directly modulate p85 subunit activity, thereby activating the PI3K/AKT axis and subsequently resulting in upregulated expression of HIF1*α* and ABCD1, concomitant with diminished lipid peroxidation and reduced ferroptosis cell death in the gastric cancer model [12]. Oxidized LDL (OX-LDL), which is recognized as a potent inducer of apoptosis in a series of models of AS in vitro, was employed for stimulating ECs and VSMCs to mimic the AS scenario, followed by RNA-Seq on the samples to validate differentially expressed genes (DEGs). The investigation further delves into their regulatory mechanisms on mTOR signaling and downstream ferroptosis and AS pathology.

The results obtained from this study contribute to our understanding of AS pathogenesis and may have implications for developing new therapeutic strategies targeting this prevalent cardiovascular disease in humans.

## 2. Materials and Methods

### 2.1. Cell Culture

Human coronary artery endothelial cells (HCAECs) were obtained from FENGHUISHENGWU (Changsha, China), Central South University (Changsha, China). The cells were cultured in RPMI 1640 medium with 10% fetal bovine serum (Gibco) and 1% penicillin/streptomycin (Solarbio, UT) at 37°C in a humidified atmosphere containing 5% carbon dioxide. HCAECs were treated with a ferroptosis agonist (Erastin, HY-15763, MCE) for 24 h at 10 *μ*M. The RPMI 1640 medium provided essential nutrients for the cells to grow and proliferate, while the fetal bovine serum supplied growth factors and hormones necessary for cell survival. The addition of penicillin/streptomycin helped prevent bacterial contamination in the culture. The incubation temperature of 37°C mimicked the physiological conditions of the human body, promoting optimal cell growth and function. The presence of 5% carbon dioxide maintained a stable pH level within the culture environment, supporting cellular metabolism and viability. HCAECs were treated with a PI3K/AKT inhibitor (BW-723C86, HY-101369, MCE) for 24 h at 10 *μ*M.

### 2.2. Plasmid Construction and Transfection

HTR2B microRNA (miRNA) is a class of endogenous eukaryotic small molecules of single-stranded RNA, usually 18–25 nt long, that can pass through binding to specific base pairs of target mRNA, causing the target sequence to fall decoy or inhibit its translation, to regulate the expression of genes after transcription control. GenePharma miRNAs use CMV promoters to express pre-miRNAs in a variety of mammalian cells, thereby achieving overexpression of target genes. The vector (miRNA) required for this control is included in this kit, which can express miRNA fragments (OE-NC) that have no homology with the target gene sequence. When the miRNA vector (OE-HTR2B) is used to transfect cells, to select the appropriate transfection method and determine the transfection efficiency, reporter genes are usually used to detect the introduction of DNA. The most commonly used reporter gene is green fluorescent protein. The miRNA expression vector provided by Shanghai Gima Company contains the expression framework of green fluorescent protein, which can express green fluorescent protein after being transferred into cells.

The transfection efficiency can be easily determined by fluorescence microscopy or flow cytometry. The products, which include HTR2B miRNA, OE-NC, OE-HTR2B, and empty carriers, were purchased from GenePharma in Shanghai, China. Transfection was performed using Lipofectamine 2000, a commonly used transfection reagent manufactured by Invitrogen Corporation in Carlsbad. The transfection process follows the instructions provided by the manufacturer to ensure efficient delivery of genetic material into the target cells.

### 2.3. Cell Counting Kit-8 (CCK-8) Assay

HCAECs cells were seeded in 48-well culture plates at a density of 5 × 10^4^ cells/well and allowed to adhere for 24 h. After exposure to different concentrations of OX-LDL (Sigma) for 48 h, the media were removed and replaced with fresh medium. Then, 10% CCK-8 solution was added to each well, and the cells were incubated at 37°C for 1 h. Following the incubation period, absorbance at 450 nm was measured using a spectrophotometer to assess cell viability and proliferation. The results were then analyzed to determine the impact of OX-LDL on HCAEC cellular activity.

### 2.4. RT-qPCR

Total RNA was extracted using TRIzol reagent (Invitrogen) followed by column-based purification with on-column DNase I digestion to remove genomic DNA; RNA quality was confirmed by NanoDrop (A260/A280:1.8-2.1; A260/A230 ≥ 2.0) and Bioanalyzer (RIN ≥ 7.0). cDNA synthesis was conducted with PrimeScript RT Master Mix (Takara) using 1 *μ*g RNA, oligo(dT), and random hexamers, with no-RT and NTC controls to exclude contamination. qPCR was performed on a CFX96 system (Bio-Rad) with PowerUp SYBR Green Master Mix (Thermo Fisher); reactions (20 *μ*L) contained 10 *μ*L master mix, 0.4 *μ*L of 10 *μ*M forward/reverse primers, 2 *μ*L diluted cDNA, and nuclease-free water. Thermal cycling included 95°C for 10 min, 40 cycles of 95°C (15 s)/60°C (1 min), and melting curve analysis. Relative expression was calculated using the 2^−*ΔΔ*Ct^ method, with reference genes validated for stability by geNorm/NormFinder; technical replicates with Ct differences > 1.5 were excluded.

### 2.5. RNA Sequencing

#### 2.5.1. RNA Sample Preparation

Biological samples were thawed on ice and immediately processed to minimize RNA degradation. For cell cultures, adherent cells were washed twice with ice-cold phosphate-buffered saline (PBS), scraped into 1 mL of ice-cold PBS, and centrifuged at 300 × g for 5 min at 4°C; the pellet was resuspended in 1 mL of TRIzol Reagent. Chloroform (200 *μ*L per 1 mL of TRIzol) was added, followed by vortexing for 15 s. The mixture was centrifuged at 12,000 × g for 15 min at 4°C. After centrifugation, the aqueous phase (containing RNA) was carefully transferred to a new RNase-free tube, avoiding the interphase and organic phase. RNA precipitation was induced by adding isopropanol (500 *μ*L per 1 mL of TRIzol), vortexing, and incubating at RT for 10 min. The sample was centrifuged at 12,000 × g for 10 min at 4°C, and the RNA pellet was washed with 75% ethanol (1 mL per 1 mL of TRIzol). After centrifugation at 7500 × g for 5 min at 4°C, the ethanol was decanted, and the pellet was air-dried for 5–10 min at RT. The RNA pellet was resuspended in 50 *μ*L of RNase-free water (prewarmed to 65°C to enhance solubility). To remove genomic DNA contamination, 1 *μ*L of DNase I (1 U/*μ*L) and 1 *μ*L of 10× DNase reaction buffer (Thermo Fisher Scientific) were added, mixed gently, and incubated at 37°C for 30 min. The reaction was terminated by adding 1 *μ*L of EDTA (0.5 M, pH 8.0) and incubating at 65°C for 10 min.

#### 2.5.2. Quality and Quantity Assessment

Qualified RNAs were quantified by Qubit 3.0 with the Qubit RNA Broad Range Assay Kit (Life Technologies, Q10210). Two microgram total RNAs were used for stranded RNA sequencing library preparation using the KCTM Stranded mRNA Library Prep Kit for Illumina, following the manufacturer's instructions. PCR products corresponding to 200–500 bps were enriched, quantified, and finally sequenced on the NovaSeq 6000 sequencer (Illumina) with the PE150 model. Raw reads containing more than 2-N bases were first discarded. Then, adaptors and low-quality bases were trimmed from raw sequencing reads using FASTX-Toolkit (Version 0.0.13). The short reads less than 16 nt were also dropped. After that, clean reads were aligned to the GRCh38 genome by HISAT2 allowing four mismatches. Uniquely mapped reads were used for gene reads number counting and FPKM calculation (fragments per kilobase of transcript per million fragments mapped).

### 2.6. Gene Ontology (GO) and Kyoto Encyclopedia of Genes and Genomes (KEGG) Pathway Enrichment Analyses

To ascertain the functional significance of 1216 DEGs identified by RNA-Seq (including 885 upregulated genes and 330 downregulated genes), functional annotation enrichment profiling was conducted using the Database for Annotation, Visualization, and Integrated Discovery (DAVID, Version 6.8; http://david.abcc.ncifcrf.gov/). This analysis encompassed GO enrichment profiling across the three primary domains: biological process (BP), molecular function (MF), and cellular component (CC). Additionally, the KEGG database was utilized to identify significantly enriched metabolic pathways within the DEG list. Statistically significant results (*p* < 0.05) from both the GO enrichment analysis and KEGG pathway analysis were subsequently visualized utilizing the bioinformatics platform (http://www.bioinformatics.com.cn/).

### 2.7. Western Blot Assay

Western blotting was performed by lysing cells in RIPA buffer (Sigma) with a complete protease inhibitor mixture on ice for 30 min. After collecting cell debris and centrifuging at 12,000 rpm for 15 min, the protein concentration was determined using a BCA protein assay kit (Beyotime, Beijing, China). Protein extracts were then separated using SDS-PAGE and transferred to PVDF membranes. The membranes were blocked with 5% skimmed milk for 2 h and incubated overnight at 4°C with antibodies targeting GPX4, SLC7A11, PI3K, p-PI3K p85, AKT, p-AKT, and *β*-actin (1:1000; Abcam, Cambridge, United Kingdom). Subsequently, the membranes were incubated with an HRP-conjugated secondary antibody (1:5000; Cell Signaling Technology) for 1.5 h at room temperature. An enhanced chemiluminescence kit (Millipore, Billerica, MA, United States) was used to visualize the blot, and protein bands were quantified using ImageJ software.

### 2.8. Malondialdehyde (MDA), 4-Hydroxynonenal (4-HNE), Glutathione (GSH), and Iron Ion Quantification

For the quantification of MDA and 4-HNE adducts, protein lysates were prepared from tissues or pelleted cells using freshly prepared RIPA buffer containing protease inhibitors. Following clarification by centrifugation (12,000 × g, 15 min, 4°C), protein concentration in the supernatant was determined via BCA assay. Equivalent protein amounts were then analyzed using the Lipid Peroxidation (MDA) Assay Kit (Colorimetric/Fluorometric, Abcam, ab118970) and the OxiSelect HNE Adduct Competitive ELISA Kit (Cell Biolabs, STA-838) according to the respective manufacturer's instructions. For measuring GSH content, briefly, harvested cells were washed with ice-cold PBS, pelleted by centrifugation (1500 × g, 5 min, 4°C), and subjected to thermal lysis through two rapid cycles of freezing in liquid nitrogen (−196°C) followed by thawing at 37°C. After equilibration at 4°C for 5 min, lysates were clarified via high-speed centrifugation (10,000 × g, 10 min, 4°C). The resulting supernatant was aliquoted, and intracellular GSH concentration was determined spectrophotometrically using the GSH and GSSG Assay Kit (Beyotime Biotechnology, S0053), strictly adhering to the kit protocol. Finally, iron ion quantification in cells and tissue homogenates employed the Iron Assay Kit (Colorimetric) (Sigma-Aldrich, MAK025). Samples were homogenized in the provided assay buffer, clarified by centrifugation, and the supernatant was analyzed according to the manufacturer's established colorimetric procedure for Fe^2+^ detection. All spectrophotometric and fluorometric measurements were performed in technical triplicates.

### 2.9. Measurement of Total Reactive Oxygen Species (ROS)

Total intracellular ROS were quantified using dihydroethidium (DHE) staining and flow cytometry. Briefly, adherent cells were detached with trypsin-EDTA, washed twice with PBS, and resuspended in complete RPMI 1640 medium (supplemented with 10% fetal bovine serum). Cell suspensions were incubated with 400 *μ*M DHE (Sigma-Aldrich, #37291) for 15 min at 37°C under a 5% CO_2_ atmosphere, with protection from light exposure. Subsequently, cells were pelleted and washed twice with prewarmed PBS, followed by resuspension in 200 *μ*L PBS. Flow cytometric analysis was performed on a minimum of 10,000 single-cell events per sample using a BD Fortessa instrument (BD Biosciences), with fluorescence detection in the phycoerythrin (PE) channel (excitation 488 nm/emission 585 nm).

### 2.10. Animals

Apolipoprotein E knockout (ApoE^−/−^) male mice, 15 in total and weighing 20 ± 2 g, were obtained from Jiangsu Jicui Pharmachem Biotechnology Co. The mice were carefully selected for their genetic background and overall health to ensure the reliability of the study. Upon arrival, they were housed in a controlled environment with regulated temperature and lighting to minimize any potential stress. The mice were divided into three groups of five each: the control group and the AS model group. The AS group is divided into the AS group and the BW-723C86 group. Mice in the BW-723C86 group received intraperitoneal injections of BW-723C86 (5 mg/kg body weight, Sigma-Aldrich) on alternate days. The AS group was treated with saline containing 0.01% DMSO. Each group was housed separately to prevent any cross-contamination or interference between the different diets. After acclimating to regular maintenance chow for a week, during which their behavior and food intake were closely monitored, the control group was given a normal diet, while the AS model group was fed a high-fat chow for 10 consecutive weeks to induce AS. Throughout this period, both groups received regular veterinary checkups to ensure their well-being and detect any potential health issues early on. The high-fat diet was carefully formulated to mimic human dietary habits associated with an increased risk of AS development, considering factors such as fat content and composition. At the endpoint of the study, all mice were euthanized following strict ethical guidelines for animal research. Tissue samples including arteries and blood samples were collected for further analysis using various techniques such as histology, immunohistochemistry, and molecular biology assays. This comprehensive approach allowed us to gain valuable insights into the mechanisms underlying AS development in ApoE^−/−^ male mice that were fed a high-fat diet.

### 2.11. Hematoxylin and Eosin (H&E) Staining

To observe changes in coronary vascular tissue morphology, we performed H&E staining. Tissue fixed with 4% paraformaldehyde was processed through decreasing alcohol concentrations, xylene washes, wax dipping, and embedding before being cut into 5-*μ*m sections. These sections were then dewaxed and stained with hematoxylin and 1% hydrochloric acid in alcohol, followed by eosin solution staining. After dehydration in alcohol and xylene washes, the sections were observed under a microscope (40× magnification; Olympus).

### 2.12. Immunohistochemistry

The coronary vascular tissue sections embedded in paraffin were deparaffinized using xylene and then rehydrated with a series of ethanol solutions ranging from 100% to 70%. After heating in EDTA for 15 min, the sections were treated with 3% H2O2 for 10 min. Subsequently, they were blocked with 10% goat serum at 37°C for half an hour. The slides were then exposed to a primary antibody against HTR2B (1:100, Proteintech, Wuhan, China) overnight at 4°C. The following day, IgG-HRP-conjugated secondary antibody (Bioss) was applied and incubated at room temperature for 1 h. This was followed by staining the samples with 3,3′-diaminobenzidine (DAB, ZSGB-Bio) and hematoxylin. Finally, the tissues were examined under a microscope and photographed (200× magnification, Olympus).

### 2.13. Statistical Analysis

The data represents the mean of three independent experiments, and numerical values are presented as standard deviation ± mean. Statistical analyses were conducted using GraphPad 7.0 software (GraphPad Software Inc., La Jolla, CA). Student's *t*-test was used to analyze differences between two groups when the data followed a normal distribution, while analysis of variance (ANOVA) was performed for three or more groups. For nonnormally distributed data between two groups, the Mann–Whitney test was employed. A *p* value less than < 0.05 indicated statistical significance.

## 3. Results

### 3.1. Cell Death From AS Induced by OX-LDL Is Associated With Ferroptosis

**Figure 1:**
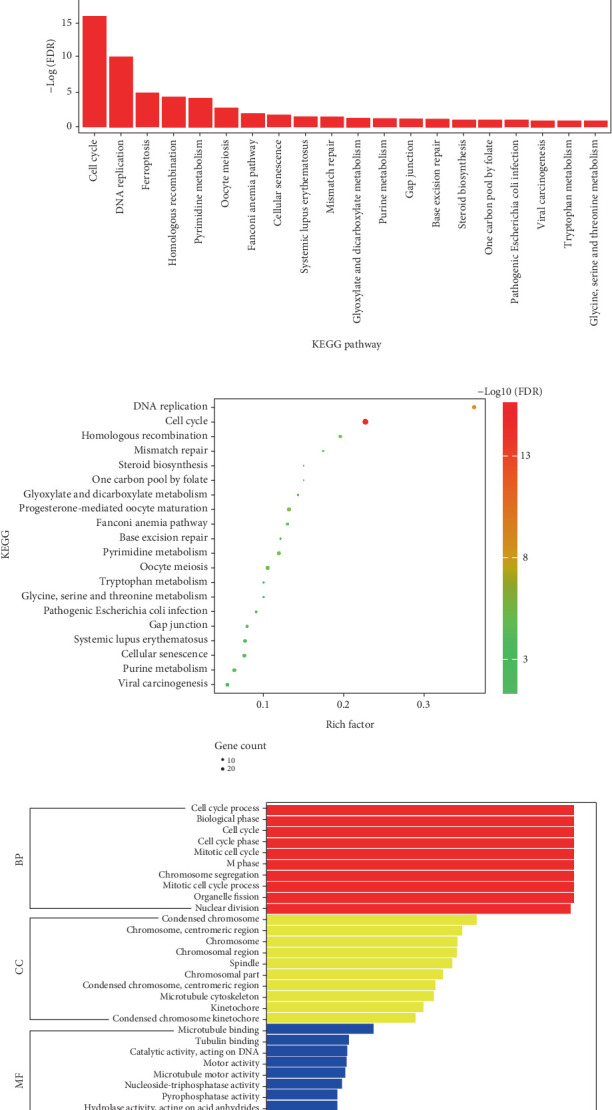
Cell modeling and sequencing analysis. (a) Relative viability of HCAECs incubated for 24 h with different concentrations of OX-LDL (*n* = 3). (b) Relative viability of HCAECs incubated with 50 *μ*g/mL of OX-LDL for 24, 48, and 72 h, respectively (*n* = 3). (c) Volcano and scatter plots of differential genes associated with coronary atherosclerosis analyzed by RNA sequencing (*n* = 3). Eight hundred and eighty-six upregulated genes and 330 downregulated genes including HTR2B and PI3KCG were shown. (d) KEGG analysis of pathway mechanisms associated with differential genes (*n* = 3). (e) GO analysis of differential gene-related pathway mechanisms (*n* = 3). ⁣^∗^*p* < 0.05, ⁣^∗∗^*p* < 0.01, and ⁣^∗∗∗^*p* < 0.001.

Our study disclosed a marked decrease in survival of HCAECs after 24-h exposure to six different concentrations of OX-LDL, followed by the cell viability detection by CCK-8 assay. The results showed that the survival of HCAECs exhibited a gradual decrease with the increase of OX-LDL concentration, and the cell survival of 75 and 100 *μ*g/mL OX-LDL-treated cells declined compared with the control group (Figure 1a). Consequently, for subsequent experiments, HCAECs were exposed to 50 *μ*g/mL OX-LDL to establish a cellular injury model. We conducted assays at various time points. It was indicated that there was a decline in HCAEC viability following 48 h of exposure to 50 *μ*g/mL OX-LDL, with decreasing survival rates correlating with extended incubation periods (Figure 1b). Furthermore, cell samples were collected from the normal control group and the selected OX-LDL-induced HCAEC model group to investigate the specific mechanism of OX-LDL on the occurrence of cellular in HCAECs. RNA-Seq analysis was conducted, and the volcano map exhibited differential genes related to AS that were conducted to screen dysregulated genes in the OX-LDL-induced HCAEC model group (Figure 1c). KEGG analysis and GO analysis showed that the OX-LDL inducing was mainly associated with DNA replication and ferroptosis (Figure 1d,e).

### 3.2. OX-LDL-Induced AS Model Enhanced Ferroptosis in HCAECs

**Figure 2:**
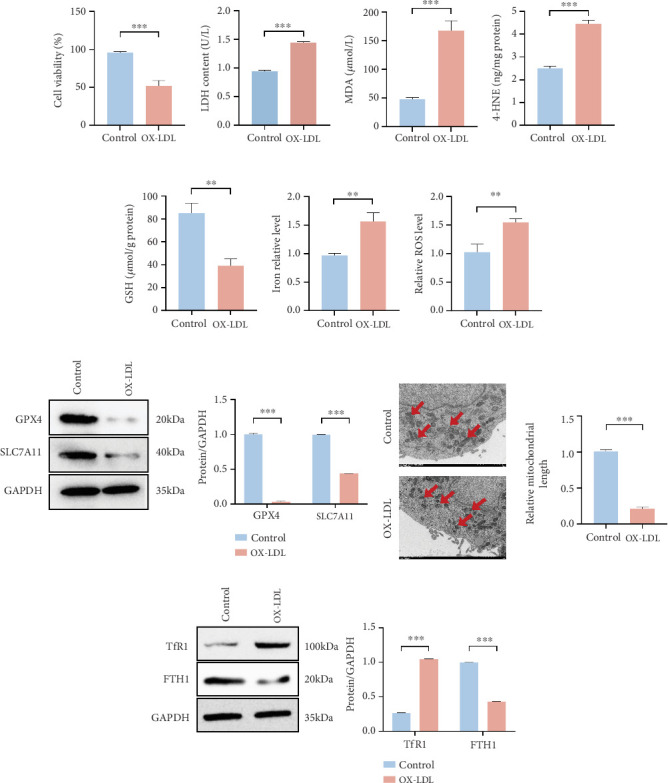
OX-LDL treatment induced ferroptosis in HCAECs. (a) Comparison of cell viability between control and OX-LDL groups. (b) LDH content in control and OX-LDL groups. (c–g) MDA, 4-HNE, GSH, iron contents, and ROS levels in control and OX-LDL groups. (h) Western blot was used to detect the expression of GPX4 and SLC7A11 proteins in both groups. (i) Transmission electron microscope was used to show mitochondrial morphology. (j) Western blot was used to detect the expression of TfR1 and FTH1 proteins in both groups. ⁣^∗∗^*p* < 0.01 and ⁣^∗∗∗^*p* < 0.001.

To further explore the effect of OX-LDL on HCAECs, the CCK-8 assay was used to detect cell viability. The results showed that cell survival was significantly reduced after OX-LDL treatment, which suggests that OX-LDL inhibited the viability of HCAECs and promoted the damage of HCAECs (Figure 2a). Based on the typical biological events of ferroptosis, we systematically evaluated the effects of OX-LDL on HCAECs. Our findings revealed that treatment with OX-LDL induced abnormal accumulation of intracellular Fe^2+^, inducing the Fenton reaction to promote the massive release of ROS, which in turn led to significant depletion of GSH. The exhaustion of GSH triggered robust activation of lipid peroxidation. The sustained accumulation of lipid peroxidation products not only disrupted the structural integrity of the cell membrane (manifested by increased release of lactate dehydrogenase, LDH) but also promoted the generation of toxic metabolites, including MDA and 4-HNE. Ultimately, these sequential pathological processes induced ferroptosis in HCAECs (Figures 2b, 2c, 2d, 2e, 2f, and 2g). To further investigate the mechanism of ferroptosis induced by OX-LDL in HCAECs, the expression of GPX4 and SLC7A11 ferroptosis-related proteins was detected by western blot. SLC7A11–GPX4 is a typical pathway system that inhibits ferroptosis and has been extensively characterized. The results of western blot showed that the expression of two proteins, SLC7A11 and GPX4, was decreased in the OX-LDL-treated HCAECs, suggesting that the physiological activity of ferroptosis was induced to occur (Figure 2h). In addition, transmission electron microscopy (TEM) revealed that mitochondrial morphology was damaged and fragmentation occurred after OX-LDL treatment (Figure 2i). To further investigate the mechanism of Fe^2+^ accumulation in HCAECs following treatment with OX-LDL, we examined the expression levels of transferrin receptor (TfR1) and ferritin Heavy Chain 1 (FTH1), which are two key iron metabolism molecules. The results demonstrated that OX-LDL stimulation significantly upregulated TfR1 expression, indicating a marked enhancement in the iron uptake capacity of HCAECs. Meanwhile, as the primary iron storage protein in cells, FTH1 expression was downregulated after OX-LDL treatment, suggesting a significant impairment of its iron storage function and consequent massive release of intracellular free iron (Figure 2j). In summary, the overactivation of TfR1 and the functional deficiency of FTH1 synergistically promoted the progression of ferroptosis.

### 3.3. HTR2B Inhibited Ferroptosis and Enhanced Cellular Activity in HCAECs

**Figure 3:**
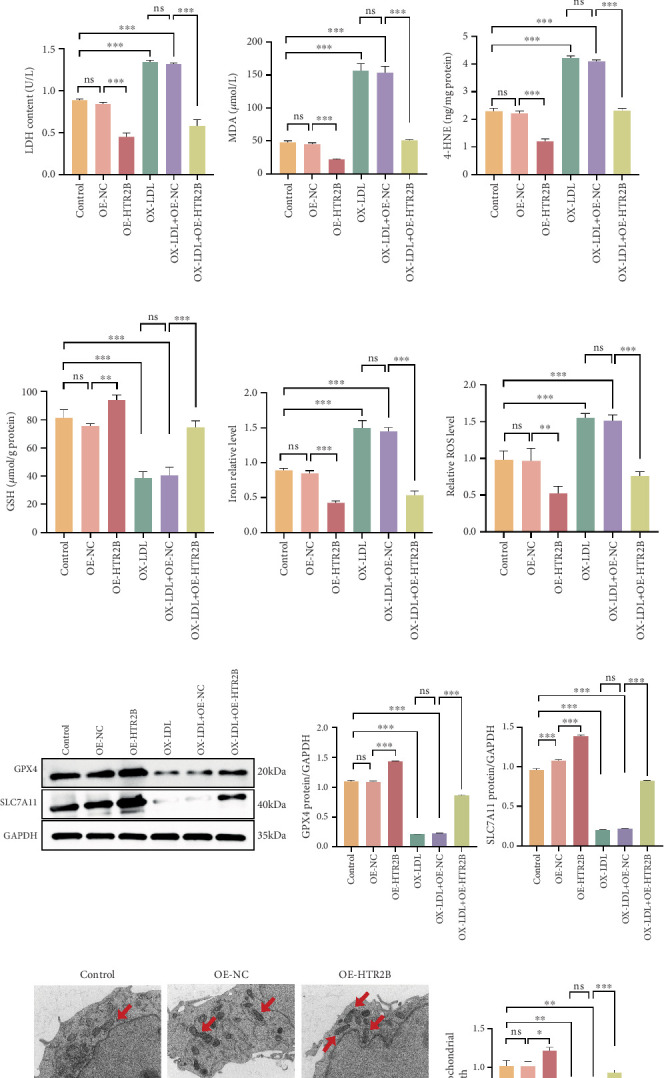
In vitro validation of the correlation between the HTR2B gene and ferroptosis. (a) RT-qPCR was performed to detect HTR2B mRNA expression levels in both groups. (b) Comparison of cell viability after different plasmid transfections. (c) LDH content was detected after different plasmid transfections. (d–h) MDA, 4-HNE, GSH, iron contents, and ROS levels were detected after different plasmid transfections. (i) Western blot was used to detect the expression of GPX4 and SLC7A11 proteins in different groups. (j) Transmission electron microscope was used to show mitochondrial morphology. ⁣^∗^*p* < 0.05, ⁣^∗∗^*p* < 0.01, and ⁣^∗∗∗^*p* < 0.001.

Research by Wenglén et al. suggests that there is a connection between the HTR2B gene and ferroptosis in VSMCs; thus, we will further validate the effect of *HTR2B* on ferroptosis of HCAECs. We first identified a decreased mRNA expression level of HTR2B in the OX-LDL group by RT-qPCR (Figure 3a). The HCAECs were transfected with HTR2B overexpression plasmids and subsequently treated with OX-LDL. CCK-8 assay showed that the survival rate was lifted in the OE-HTR2B + OX-LDL group versus the OX-LDL group and was close to the survival rate of the untreated group. However, in the blank plasmid group (OE-NC + OX-LDL), cell survival remained low, suggesting a link between the HTR2B gene and cellular ferroptosis (Figure 3b). Similarly, groups of cells were collected and assayed for LDH, MDA, 4-HNE, GSH, iron content, and ROS level. The results showed significant decreases in LDH, MDA, 4-HNE, iron, and ROS levels, whereas an upregulation of GSH content was observed in the HTR2B overexpression group (Figures 3c, 3d, 3e, 3f, 3g, and 3h). Western blot results showed corresponding results with significant upregulation of the expression of SLC7A11 and GPX4 in the OE-HTR2B + OX-LDL-treated group, suggesting inhibition of the physiological process of ferroptosis. The expression of two proteins, SLC7A11 and GPX4, was significantly downregulated in the OE-NC + OX-LDL-treated group, suggesting that the physiological process of ferroptosis occurs normally and that the expression of the corresponding proteins was reduced (Figure 3i). Similarly, HTR2B also alleviated mitochondrial damage caused by OX-LDL, and mitochondrial fragmentation was reduced in the HTR2B overexpression group (Figure 3j). The above results suggest that the HTR2B gene reduces the occurrence of cellular ferroptosis in HCAECs.

### 3.4. HTR2B Inhibited Ferroptosis via Activating PI3K/AKT Axis

**Figure 4:**
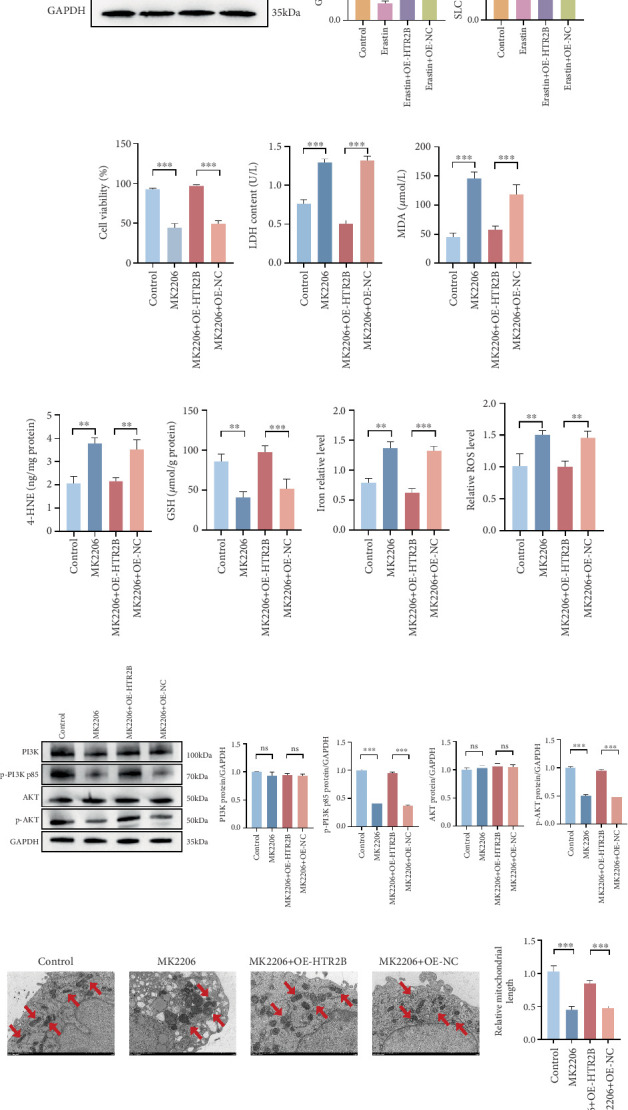
In vitro probing of ferroptosis mechanisms and downstream molecular pathway exploration. (a) Western blot detection of ferroptosis agonist downregulates the expression of two proteins, SLC7A11 and GPX4, with grayscale analysis. (b–h) Effects of PI3K/AKT pathway inhibitor on ferroptosis mechanisms (cell viability, LDH, MDA, 4-HNE, GSH, iron content, and ROS level). (i) Images and relative gray value analysis of western blot for detection of the PI3K/Akt pathway. (j) Transmission electron microscope was used to show mitochondrial morphology. ⁣^∗∗^*p* < 0.01 and ⁣^∗∗∗^*p* < 0.001.

**Figure 5:**
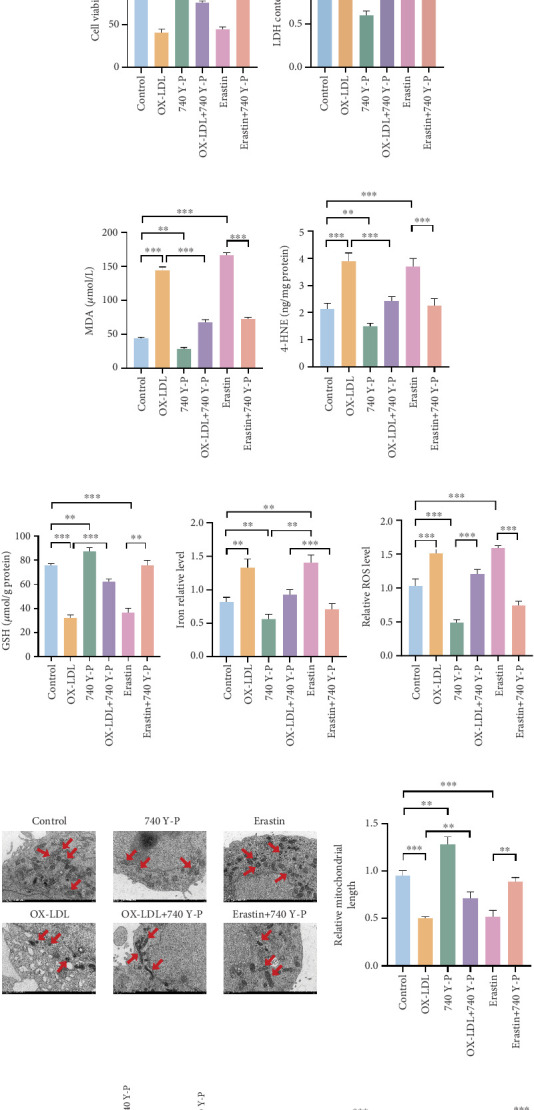
The PI3K agonist attenuates ferroptosis induced by OX-LDL. (a) Western blot detection of the PI3K/Akt pathway, with grayscale analysis. (b–h) Effects of the PI3K/AKT pathway agonist on ferroptosis mechanisms (cell viability, LDH, MDA, 4-HNE, GSH, iron content, and ROS level). (i) Transmission electron microscope was used to show mitochondrial morphology. (j) Images and relative gray value analysis of western blot for detection of the SLC7A11/GPX4 axis. ⁣^∗^*p* < 0.05, ⁣^∗∗^*p* < 0.01, and ⁣^∗∗∗^*p* < 0.001.

HTR2B dysregulation and its underlying mechanism in ferroptosis needed further validation; subsequently, we tested this hypothesis in HCAECs using ferroptosis agonists (Erastin). The results showed that the expression of SLC7A11 and GPX4 was significantly downregulated after the use of Erastin, suggesting that ferroptosis was promoted. Whereas with OE-NC, Erastin still had the effect of promoting the occurrence of ferroptosis. When Erastin was used together with OE-HTR2B, the phenotype was restored to the same as that of control, indicating that *HTR2B* could inhibit the process of ferroptosis (Figure 4). The PI3K/AKT pathway has recently been shown to be associated with ferroptosis. It has been shown that inhibition of the PI3K/AKT pathway promotes ferroptosis. To gain a deeper understanding of how *HTR2B* regulates downstream signaling pathways, we first treated cells with PI3K/AKT pathway inhibitors (MK2206), and the results showed a decrease in cell survival of HCAECs after treatment with the inhibitors, indicating that PI3K/AKT can inhibit ferroptosis (Figure 4). Cells from different groups were collected to determine LDH, MDA, 4-HNE, GSH, iron content, and ROS level. The results showed that LDH, MDA, 4-HNE, Fe^2+^, and ROS were significantly elevated and GSH content was downregulated in the MK2206 and MK2206 + OE-NC treatment groups, suggesting the promotion of ferroptosis (Figures 4, 4, 4, 4, 4, and 4). Western blot results further validated the correlation between the PI3K/AKT pathway and ferroptosis (Figure 4). After treatment with PI3K/AKT pathway inhibitors, the results showed decreased phosphorylation of PI3K and AKT-related proteins. The inhibition of the PI3K/AKT pathway also improved mitochondrial damage and fragmentation (Figure 4). These results are consistent with our previous RNA-Seq data and references, suggesting a PI3K/AKT regulatory process between *HTR2B* and ferroptosis, which further indicates that *HTR2B* can inhibit the process of ferroptosis through the PI3K/AKT pathway. To comprehensively elucidate the regulatory relationship between the PI3K/AKT signaling pathway and ferroptosis within the cellular model of AS, we conducted intervention experiments using OX-LDL, Erastin, and the PI3K/AKT pathway-specific agonist (740 Y-P). Our results revealed that OX-LDL treatment significantly suppressed PI3K/AKT pathway activity, an inhibitory effect that was effectively reversed by subsequent addition of 740 Y-P. Notably, the induction of ferroptosis via Erastin treatment also led to a marked downregulation of PI3K/AKT pathway activation. Consistently, 740 Y-P intervention alleviated ferroptosis induced by OX-LDL or Erastin in HCAECs, as evidenced by enhanced cell viability and mitigated lipid peroxidation and mitochondrial structural damage (Figures 5, 5, 5, 5, 5, 5, 5, 5, 5, and 5). Collectively, these findings demonstrated that HTR2B suppressed ferroptosis in HCAECs through the activation of the PI3K/AKT signaling pathway, thereby exerting protective effects against AS progression.

### 3.5. Inhibiting HTR2B Negated PI3K/AKT Pathway in AS Mice

**Figure 6:**
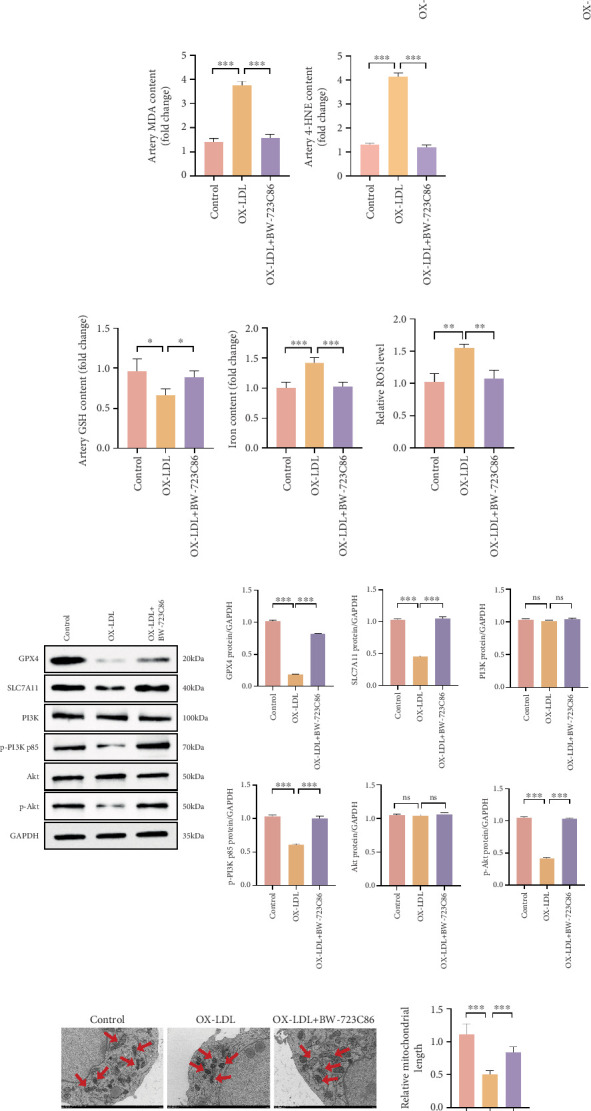
HTR2B inhibits OX-LDL-induced ferroptosis in vivo. (a) H&E staining, TUNEL staining, and HTR2B immunofluorescence assay of cross-sections of coronary vascular tissues from different groups. (b–f) MDA, 4-HNE, GSH, iron contents, and ROS levels were detected in different groups of coronary vascular tissues. (g) Western blot detection of the PI3K/AKT pathway and SLC7A11/GPX4 axis in coronary vascular tissues of different groups with grayscale analysis (*n* = 3). (h) Transmission electron microscope was used to show mitochondrial morphology. ⁣^∗^*p* < 0.05, ⁣^∗∗^*p* < 0.01, and ⁣^∗∗∗^*p* < 0.001.

**Figure 7:**
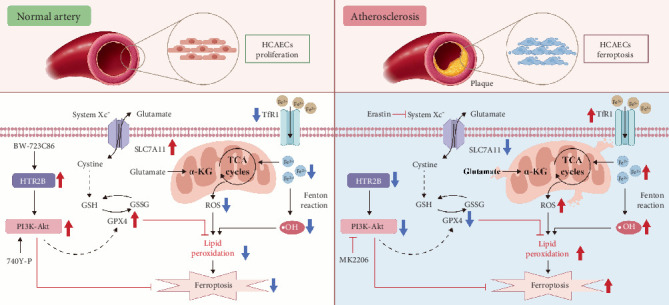
HTR2B improves AS by inhibiting HCAEC ferroptosis through the PI3K/AKT pathway.

HTR2B plays a critical role in cell viability and proliferation [11, 13–15]. To validate the hypothesis, mice with or without OX-LDL-induced AS were intraperitoneally injected with BW-723C86, a selective HTR2B agonist (5 mg/kg body weight, every other days), in which we examined the activation of the relevant pathways. Cross-sections of coronary vascular tissue were collected for H&E staining, and IF was used for TUNEL staining and detection of HTR2B protein. The results showed that after OX-LDL treatment, the expression of HTR2B was downregulated, the arrangement of VSMCs was disordered, and apoptosis increased. However, BW-723C86 reversed these effects after treatment (Figure 6a). In addition, the activation of HTR2B also promoted increased phosphorylation of PI3K/AKT in mice, further inhibiting iron ion accumulation, lipid peroxidation, and mitochondrial damage and inhibiting the occurrence of ferroptosis in vivo (Figures 6b, 6c, 6d, 6e, 6f, 6g, and 6h). Thus, we proved that HTR2B is involved in regulating the process of EC ferroptosis in vivo through the PI3K/AKT pathway and plays a role in the treatment of AS (Figure 7).

## 4. Discussion

AS is the formation of fibrofatty lesions in the arterial wall that cause much morbidity and mortality worldwide, including most myocardial infarctions and many strokes, as well as disabling peripheral arterial disease, which is characterized by increased lipid accumulation and inflammatory cytokines in the vessel wall, and dysfunction of endothelial and VSMCs [16–18]. Interestingly, ferroptosis is an iron-dependent lipid peroxidation. Elevated lysosomal activity can lead to the accumulation of intracellular iron ions and lipid peroxides. In AS, ferroptosis plays an important role in lesion progression and stability. Macrophages within plaques undergoing ferroptosis release DAMPs, influencing inflammation and potentially plaque rupture. Therapeutically targeting ferroptosis pathways, such as GPX4 inhibition or glutamate–cysteine ligase activation, offers novel strategies for AS intervention. Further research elucidating the specific mechanisms and clinical implications is warranted. Therefore, the present study established an OX-LDL-induced HCAEC model, after which we performed total RNA-Seq on HCAECs before and after stimulation with OX-LDL and combined the sequence data with the GEO database; therefore, the results are highly reliable. Interestingly, GO illuminated that genes were enriched in the ferroptosis-related axis. And among the differently expressed genes, *HTR2B* was selected for the forthcoming study after excluding known genes and those that have been well studied.

Some previous studies have utilized OX-LDL stimulation to modify the AS model, and its efficacy has been illuminated [19–21]. In the previous study, OX-LDL stimulates ECs to produce an inflammatory response. Under the stimulation of OX-LDL at a concentration of 50 *μ*g/mL, oxidative stress occurred in ECs, and ROS content increased. Meanwhile, the level of apoptosis was increased, and the secretion of inflammatory factors was increased [22]. We found that after HCAECs in human coronary artery ECs were treated with OX-LDL, significant differences in the HTR2B gene were detected by RNA sequencing analysis. In addition, OX-LDL treatment decreased cell viability and GSH levels and increased MDA levels and significant cell apoptosis. The WB results demonstrate that this cell death could occur through an induced ferroptosis mechanism. EC lines with overexpression of *HTR2B* were constructed using miRNA plasmids. The results showed that after overexpression of the HTR2B gene, the survival rate of the cells increased, the MDA expression level decreased, ferroptosis received inhibition, and the apoptosis rate decreased. The relationship between the HTR2B gene and ferroptosis was further validated using ferroptosis agonists. The expression of two proteins, SLC7A11 and GPX4, underwent downregulation after the use of ferroptosis agonists, suggesting that ferroptosis was promoted. While the ferroptosis agonist was combined with the overexpression of *HTR2B*, the expression of ferroptosis-related proteins could be restored, indicating that the overexpression of *HTR2B* plays a role in inhibiting ferroptosis.

In PI3K/AKT signaling, AKT is phosphorylated and activated after the production of PIP3. Activated AKT phosphorylates and inhibits several negative regulators of ferroptosis, including PTEN and NRF2. PTEN dephosphorylates PIP3 back to PIP2, while NRF2 regulates the expression of antioxidant enzymes and ferroptosis inhibitors such as GPX4. Thereby, the activation of PI3K/AKT might suppress ferroptosis; meanwhile, PI3K/AKT signaling downregulates the expression of ferroprotein, an iron exporter, thereby increasing intracellular iron levels and promoting lipid peroxidation. So far, several previous studies have investigated the role of PI3K signaling on ferroptosis: Yi et al. reveal that the carcinogenic activation of the PI3K-AKT-mTORC1 pathway inhibits iron death in cancer cells through downstream SREBP1/SCD1-mediated lipogenesis in a breast cancer model [23]. Nevertheless, Sun et al. uncover that LAP promotes oxidative stress through inhibiting PI3K/AKT and promoting mitochondrial dysfunction, which further induces the ferroptosis in cardiomyocytes [24]. In the current study, we first treated cells with an inhibitor of the PI3K/AKT/mTOR signaling pathway (MK2206), and the results showed that the phosphorylation level of PI3K and AKT was decreased; at the same time, PI3K/AKT inhibition decreased cell viability and GSH levels in OX-LDL-induced HCAEC model. The overexpression of HTR2B can compensate for the level of PI3K/AKT phosphorylation, indicating that its effect is opposite to that of MK2206 inhibitors, which implies that HTR2B exhibits a ferroptosis-suppressing effect via promoting PI3K/AKT signaling. However, the regulatory mechanism of HTR2B on PI3K/AKT signaling needs further investigation. Two previous studies elucidate that HTR2B binds to Fyn, regulates p85 activity, and triggers the PI3K/AKT/mTOR signaling pathway: Zhang et al. state that Fyn participates in the activation of PI3K/AKT signaling in uveal melanoma pathogenesis [25]. Tu et al. reveal that when further exploring the protective effect of HTR2B on the PI3K/AKT pathway, it was found that the classical PKC activator PMA of PI3K could not mimic the protective effect of HTR2B on metabolic stress. It is reported that HTR2B combines with Fyn; meanwhile, Fyn binds to the p85 subunit of PI3K to activate PI3K/AKT signaling [12].

Finally, the relationship between HTR2B and AS was verified in vivo. We demonstrated the activation of the pathway in vivo through ApoE^−/−^ animal models and treatment with a selective HTR2B agonist (BW-723C86). The results showed that the expression of related proteins in the PI3K-AKT signaling pathway was reduced in the experimental group, suggesting that the experimental group induced the onset of the ferroptosis process, indicating that blocking the expression of HTR2B can accelerate the ferroptosis of cells in vivo. Sections of cross-sections of vascular tissue illustrated that ECs in the experimental group showed cell death, but HTR2B could be involved in regulating the level of ferroptosis in ECs to achieve alleviation of AS.

## 5. Conclusion

Collectively, we explored *HTR2B* as a novel biomarker of HCAEC ferroptosis in AS by combining the result of RNA-Seq with the GEO database, then validated its suppressive role on ferroptosis via promoting PI3K/AKT signaling, suggesting that targeting *HTR2B* may offer a promising strategy for AS management.

## Data Availability

The data that support the findings of this study are available from the corresponding author upon reasonable request.

## Supporting Information

Supporting Information Additional supporting information can be found online in the Supporting Information section. Tables S1–S8 list all the proteins and their corresponding control blots in the western blotting experiments.
