# Supplementary material for: HTR2B-Mediated Endothelial Protection: Modulating Ferroptosis via the PI3K/AKT Signaling Pathway in Atherosclerosis
Source: Cardiovasc Ther. 2025 Oct 3;2025:7934590. doi: 10.1155/cdr/7934590 (PMC12513789; doi:10.1155/cdr/7934590)
Supplement: Supporting Information — Additional supporting information can be found online in the Supporting Information section. Tables S1–S8 list all the proteins and their corresponding control blots in the western blotting experiments. [file 7934590.f1.docx]

**Supplementary Materials: *Cardiovascular Therapeutics Journal*
Tittle:** HTR2B-Mediated Endothelial Protection: Modulating Ferroptosis via the PI3K/AKT Signaling Pathway in Atherosclerosis

**Table S1-S8** list all the proteins and their corresponding control blots in the Western blotting experiments.

**Table S1.** Figure 2G presents representative Western blots demonstrating the expression levels of GPX4 and SLC7A11, along with their corresponding loading control GAPDH, in HCAECs following OX-LDL treatment.

|  | Control | OX-LDL |
| --- | --- | --- |
| GPX4 | 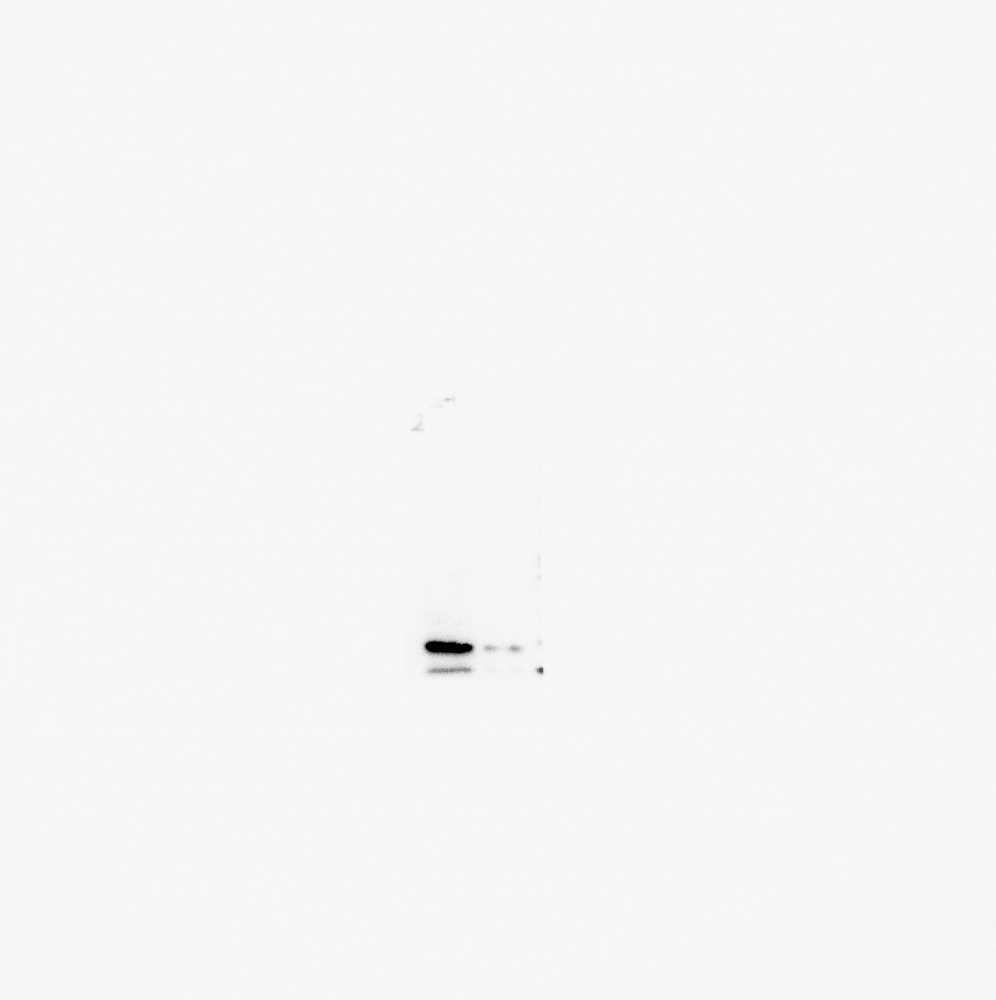 | |
| GAPDH | 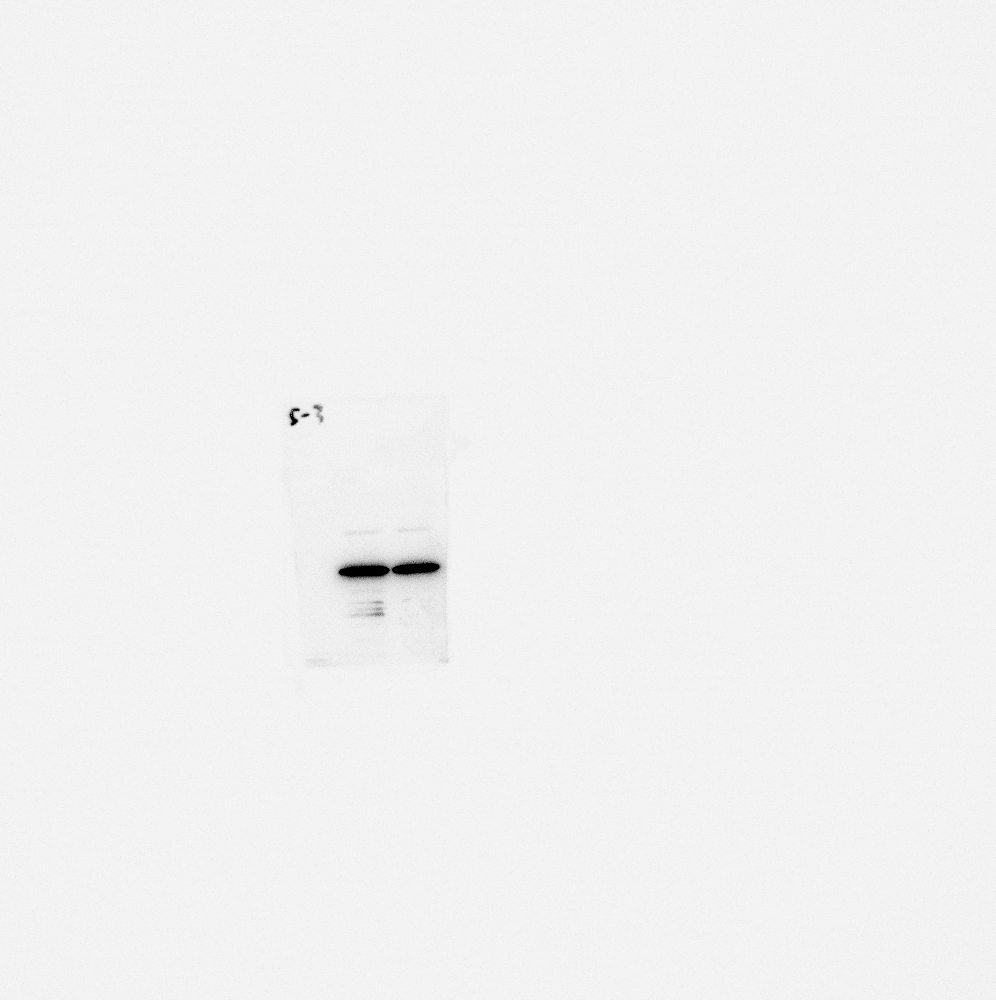 | |
| SLC7A11 | 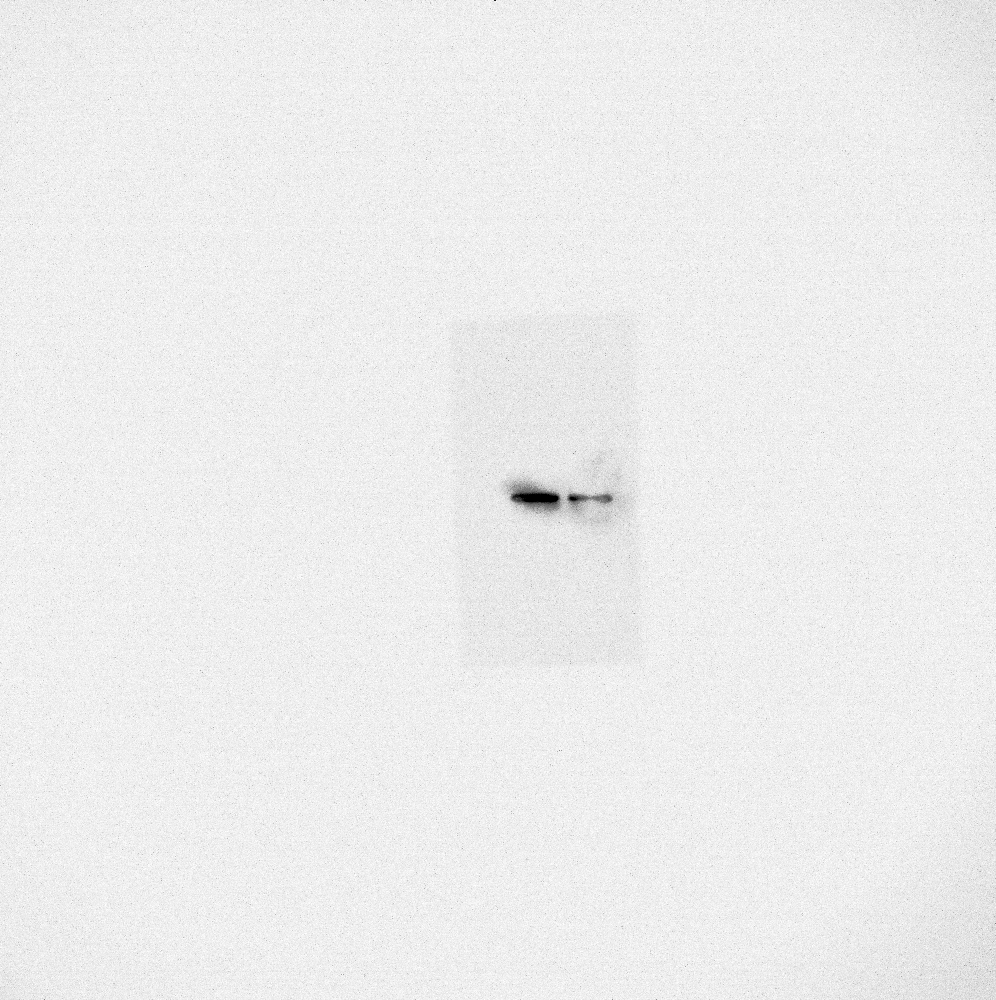 | |
| GAPDH | 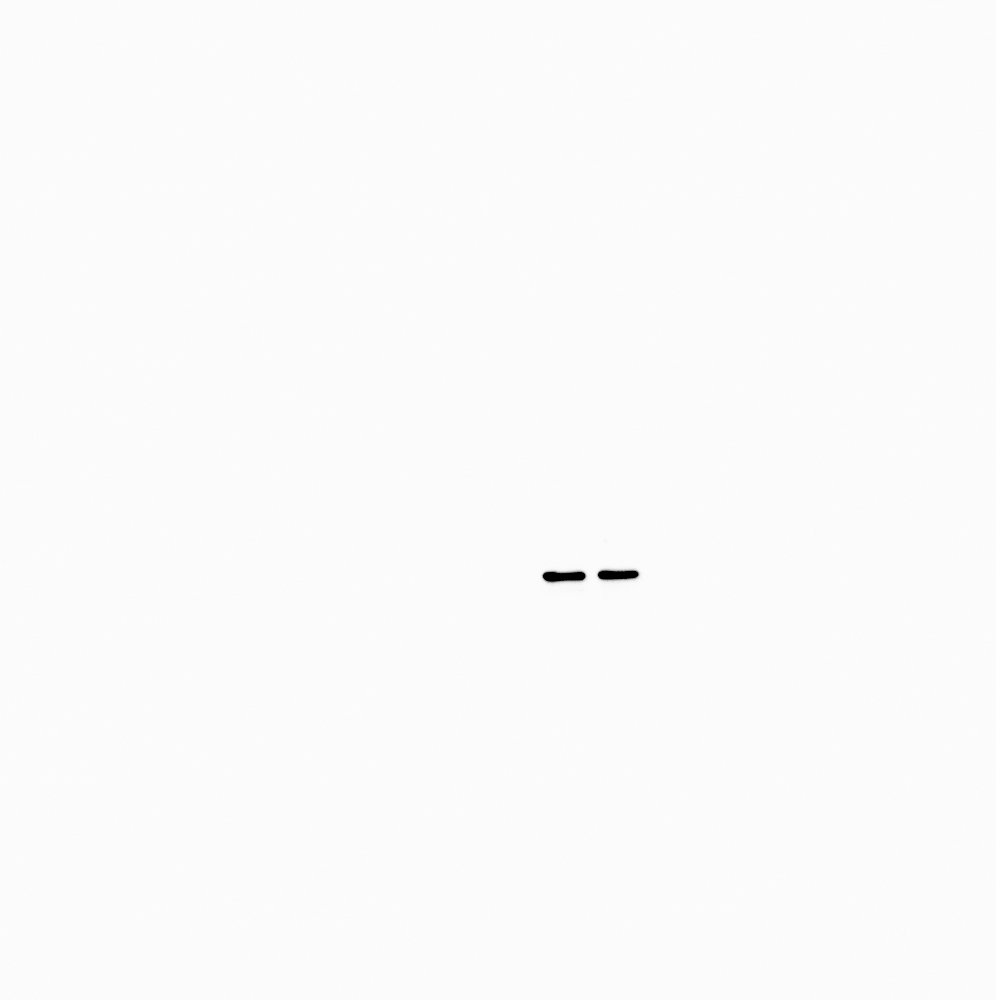 | |

**Table S2.** Figure 2I presents representative Western blots demonstrating the expression levels of TfR1 and FTH1, along with their corresponding loading control GAPDH, in HCAECs following OX-LDL treatment.

|  | Control | OX-LDL |
| --- | --- | --- |
| TfR1 | 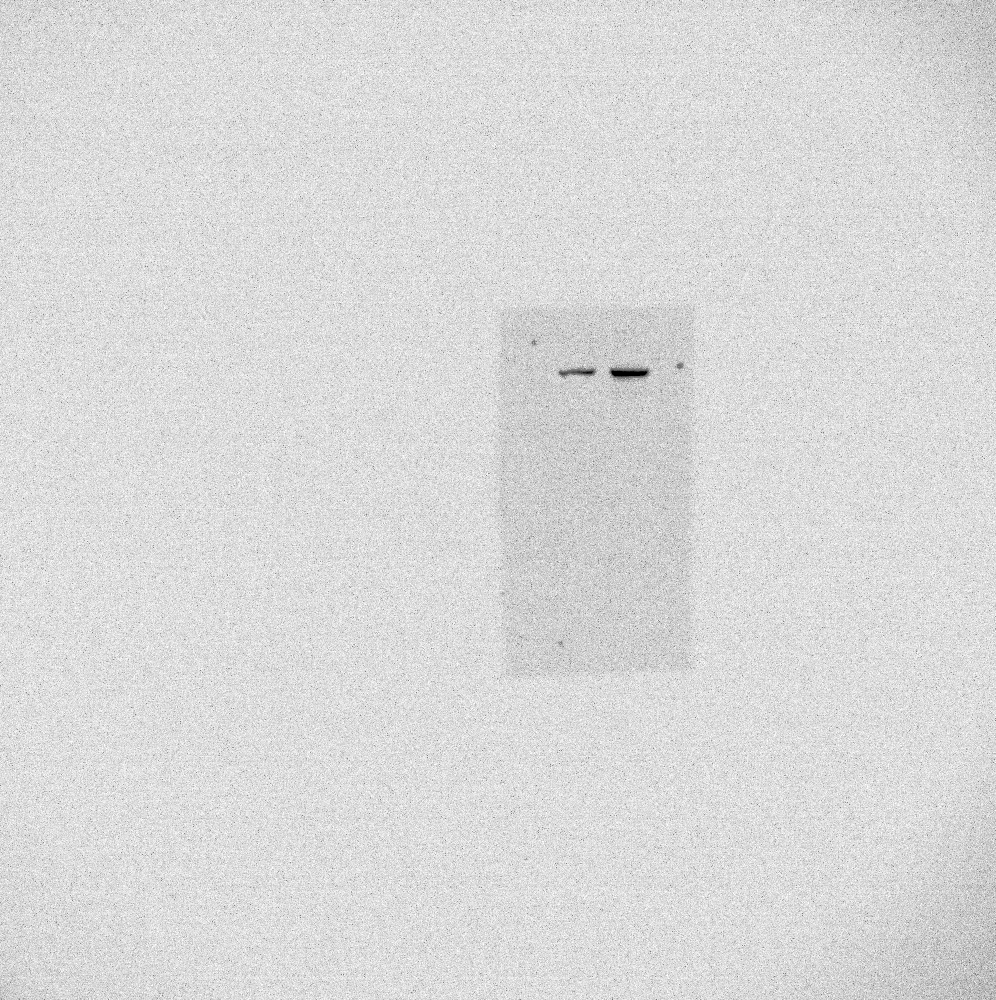 | |
| GAPDH | 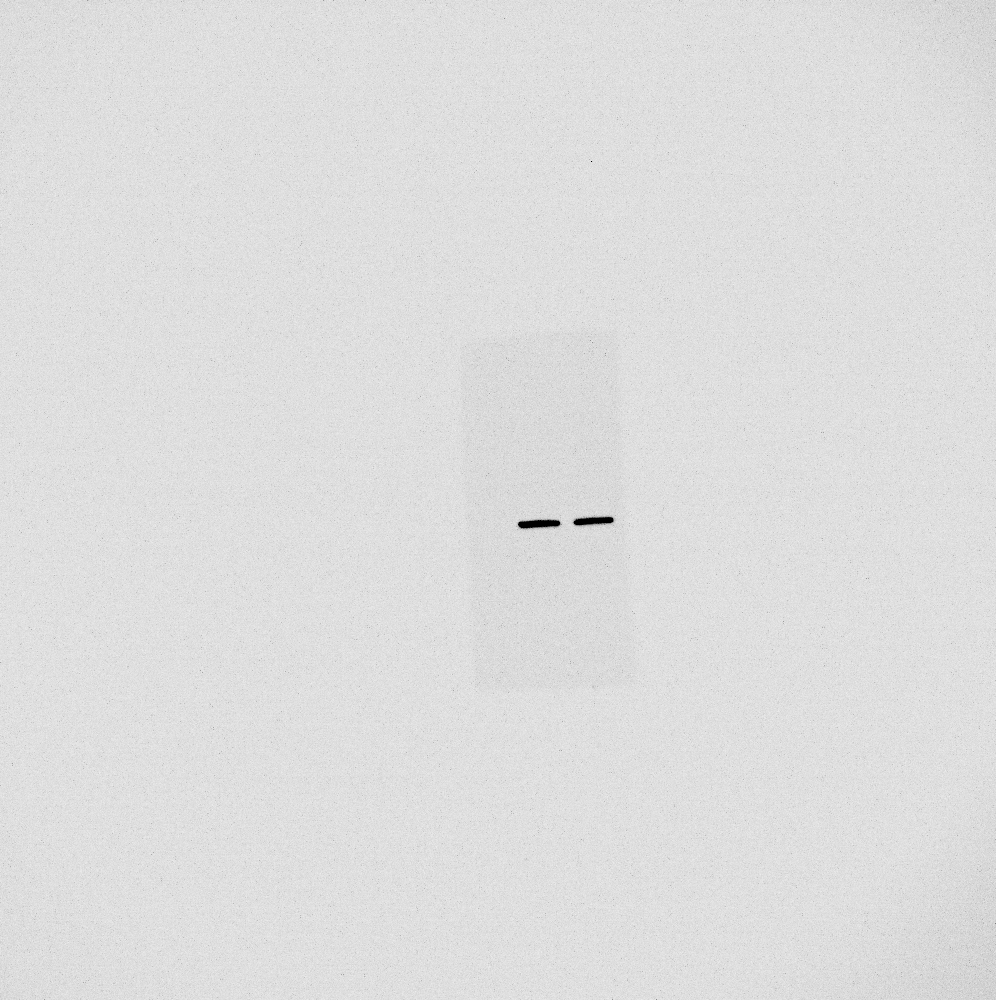 | |
| FTH1 | 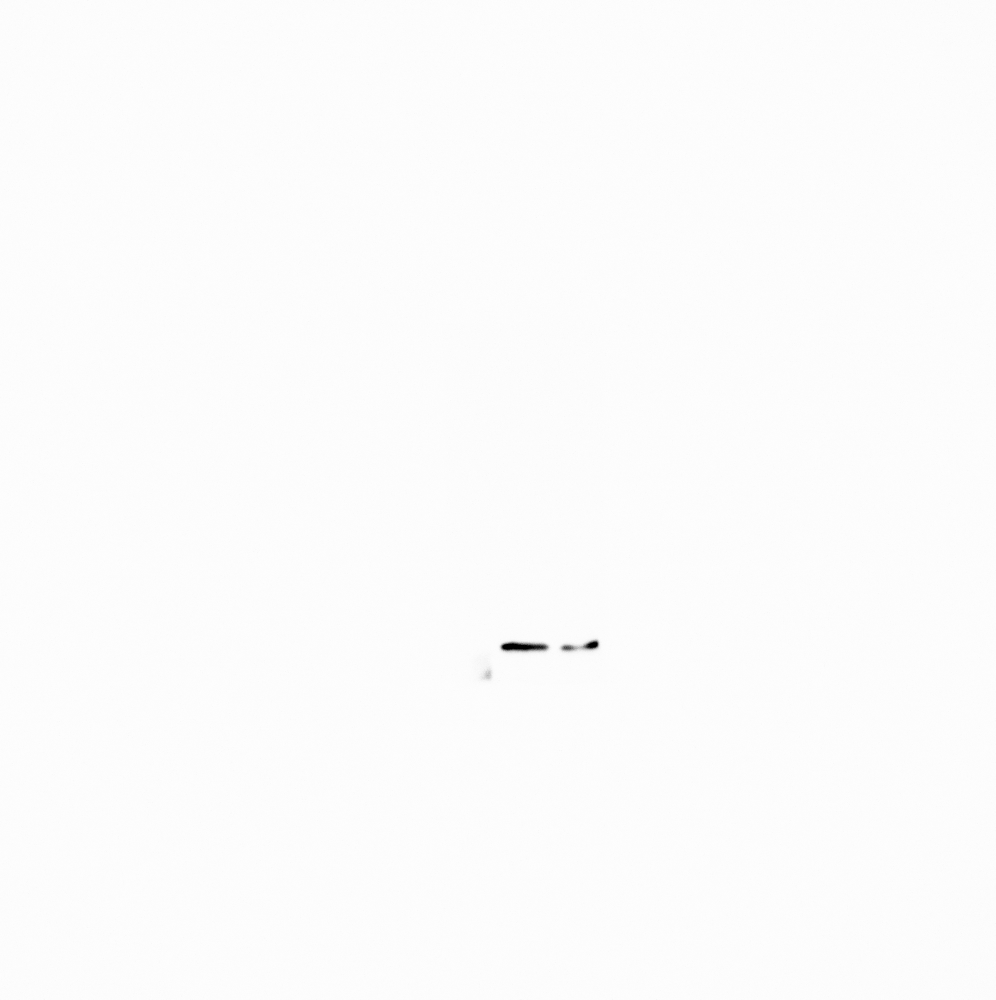 | |
| GAPDH | 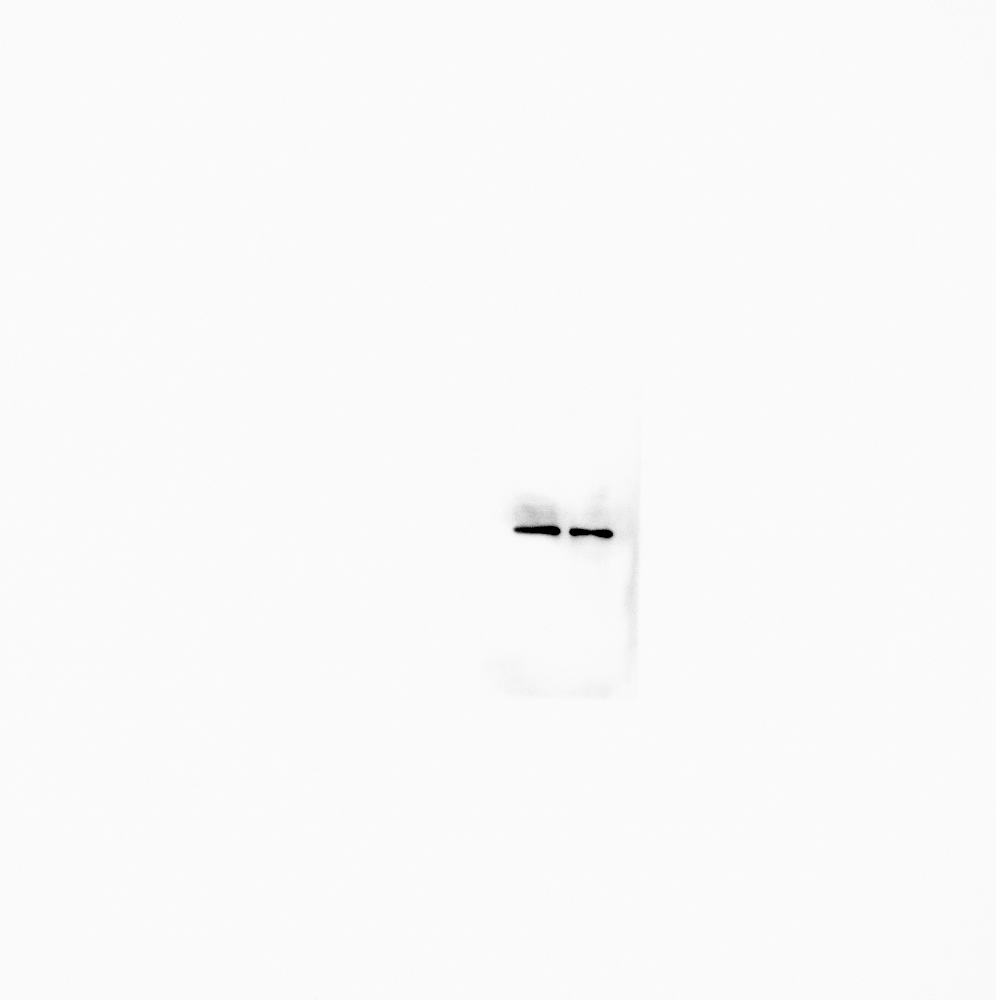 | |

**Table S3.** Figure 3H displays representative Western blots depicting GPX4 and SLC7A11 expression, along with their corresponding loading control GAPDH, in HTR2B-overexpressing HCAECs following OX-LDL treatment.

|  | Control | OE-NC | OE-HTR2B | OX-LDL | OX-LDL+OE-NC | OX-LDL+OE-HTR2B |  |
| --- | --- | --- | --- | --- | --- | --- | --- |
| GPX4 | 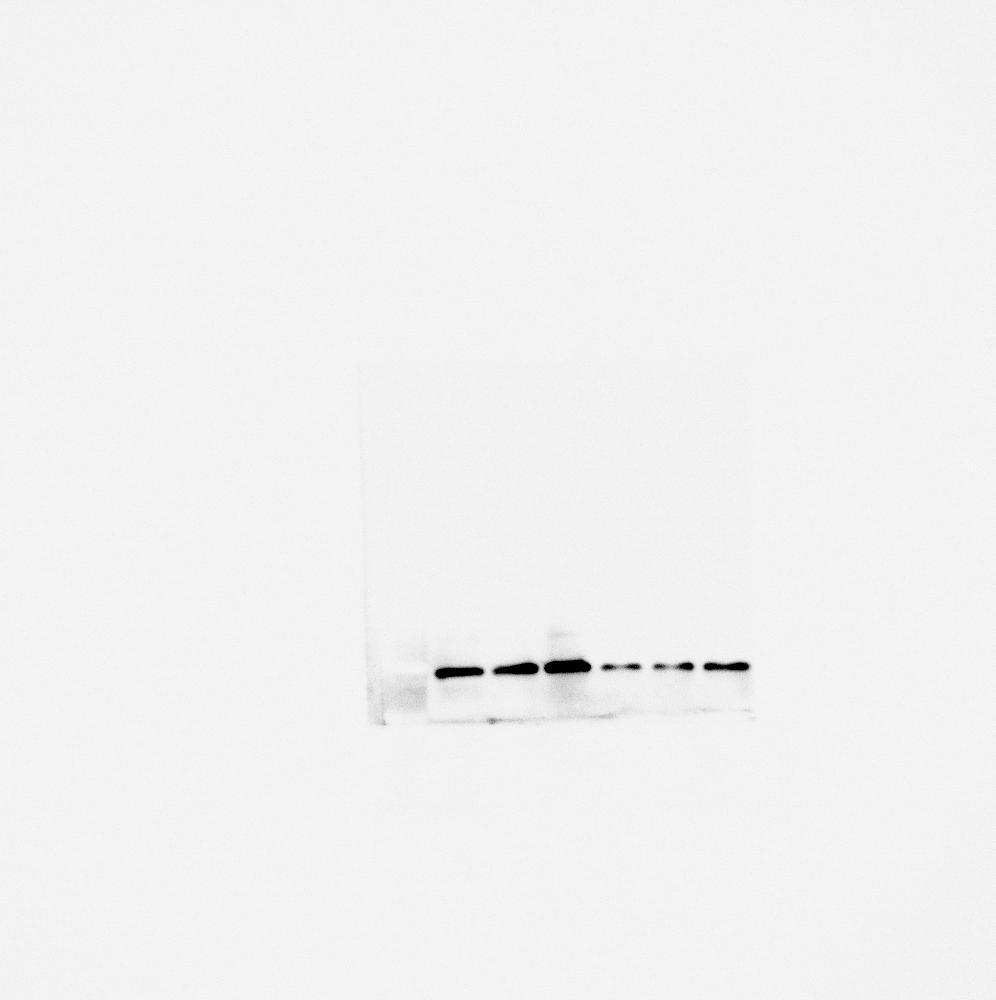 | | | | | | |
| GAPDH | 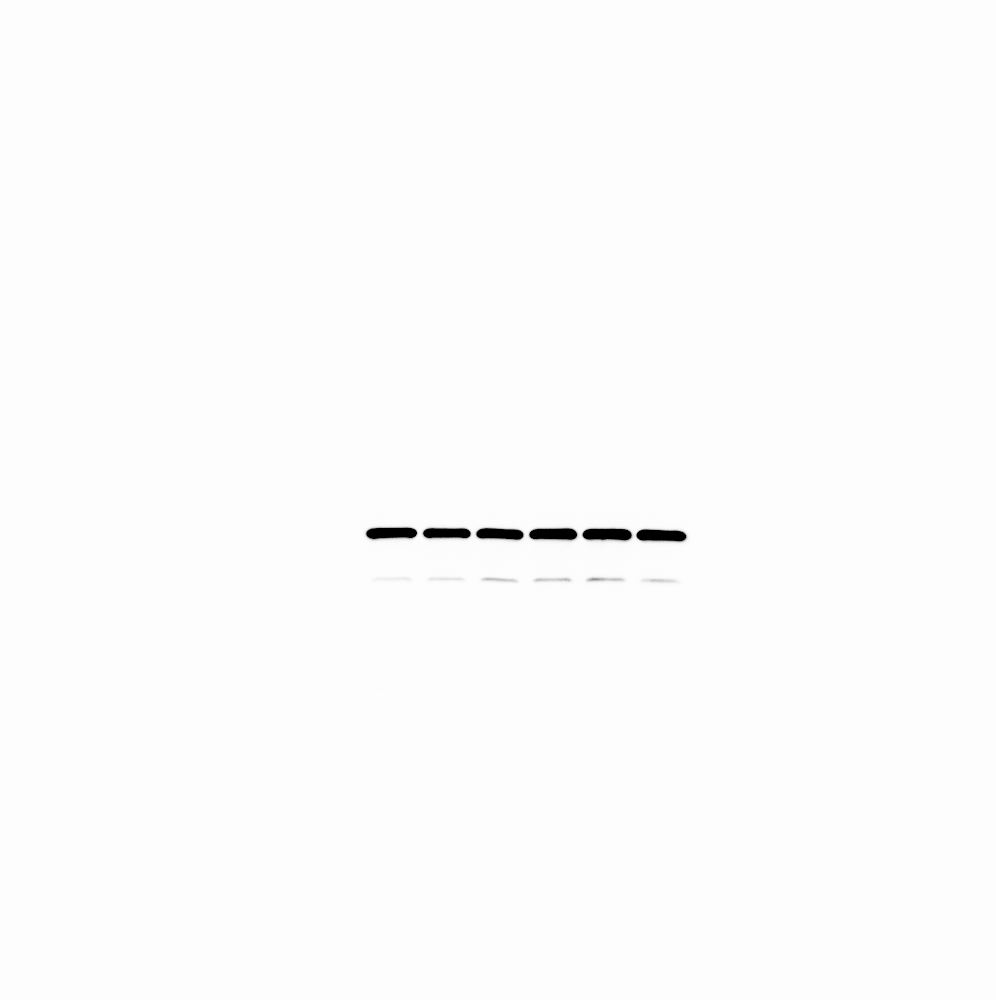 | | | | | | |
| SLC7A11 | 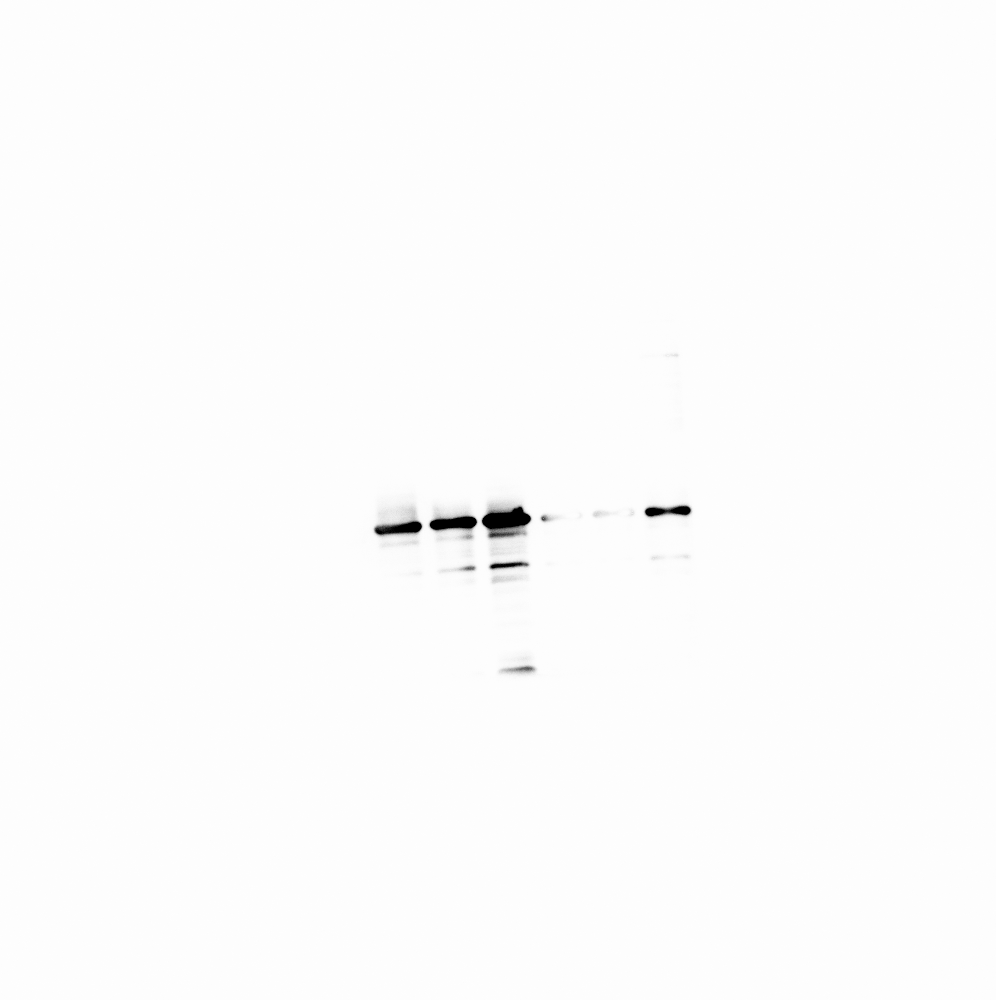 | | | | | | |
| GAPDH | 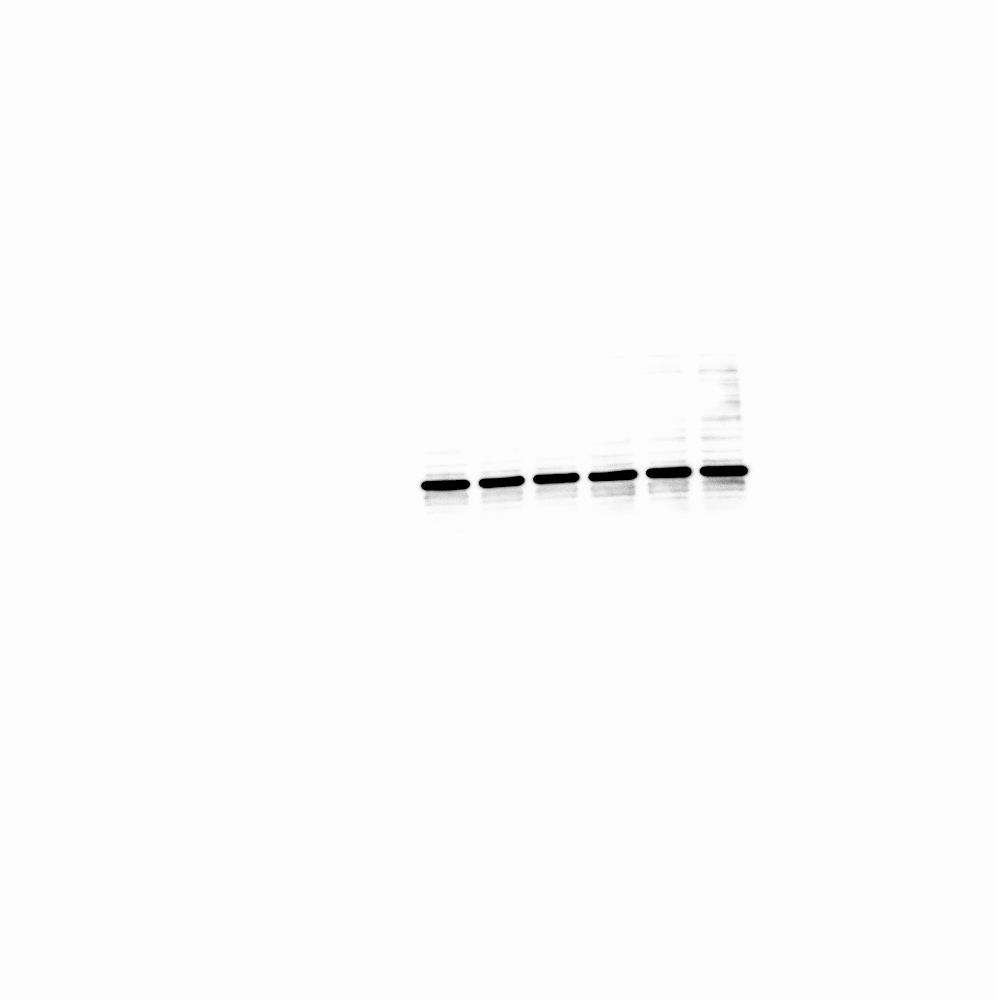 | | | | | | |

**Table S4.** Figure 4A displays representative Western blots depicting GPX4 and SLC7A11 expression, along with their corresponding loading control GAPDH, in HTR2B-overexpressing HCAECs following Erastin treatment.

|  | Control | Erastin | Erastin+OE-HTR2B | Erastin+OE-NC |
| --- | --- | --- | --- | --- |
| GPX4 | 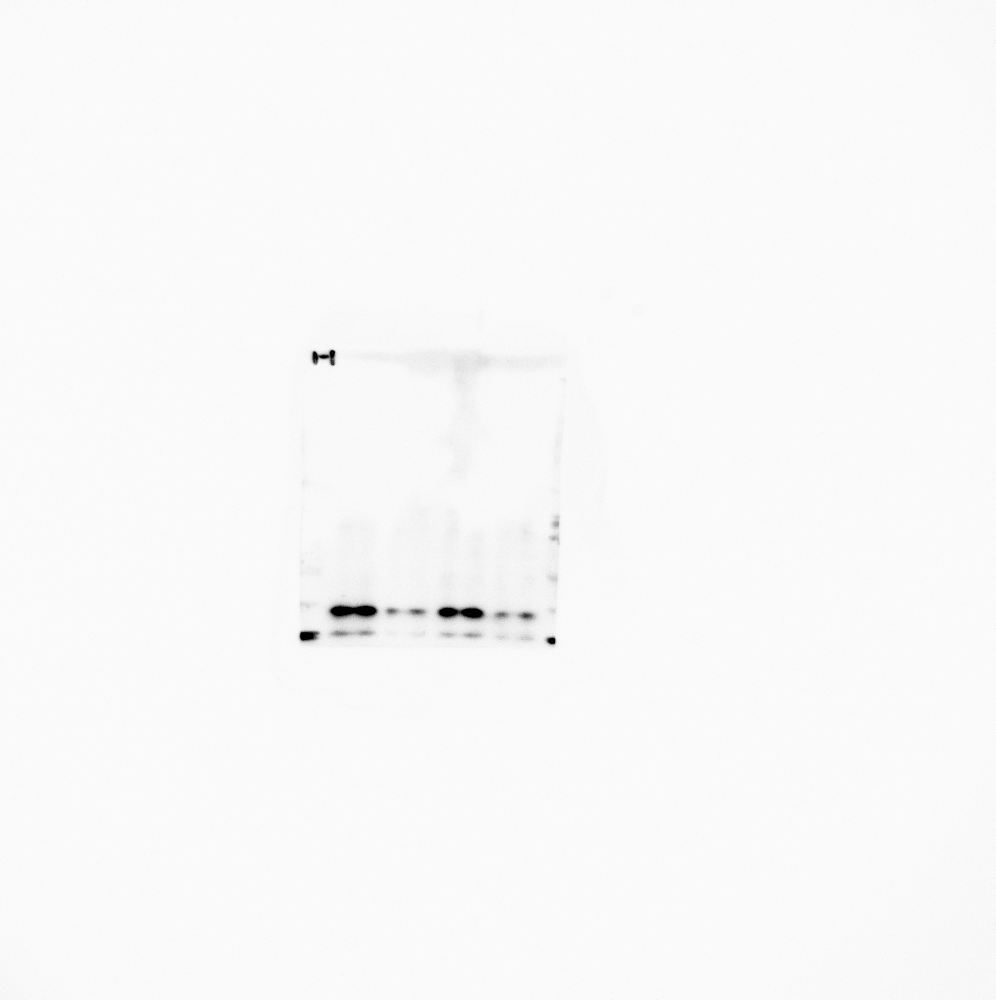 | | | |
| GAPDH | 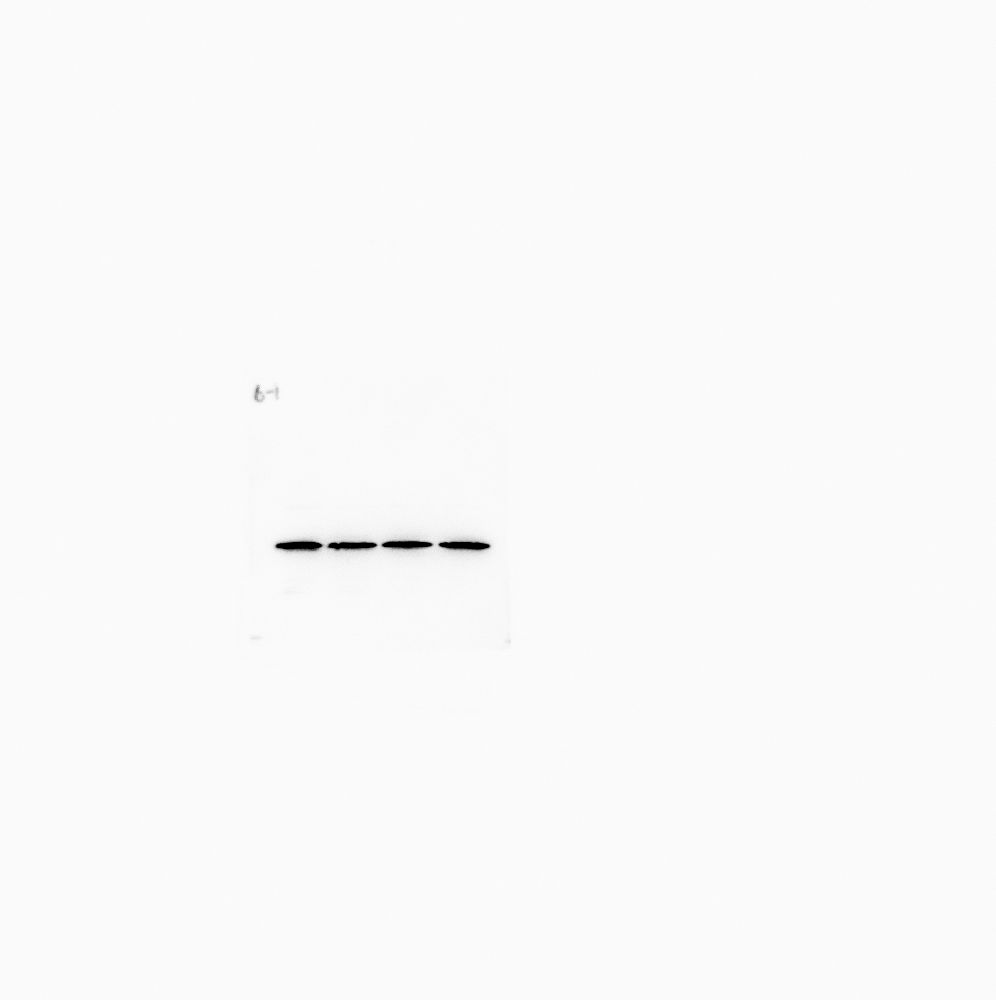 | | | |
| SLC7A11 | 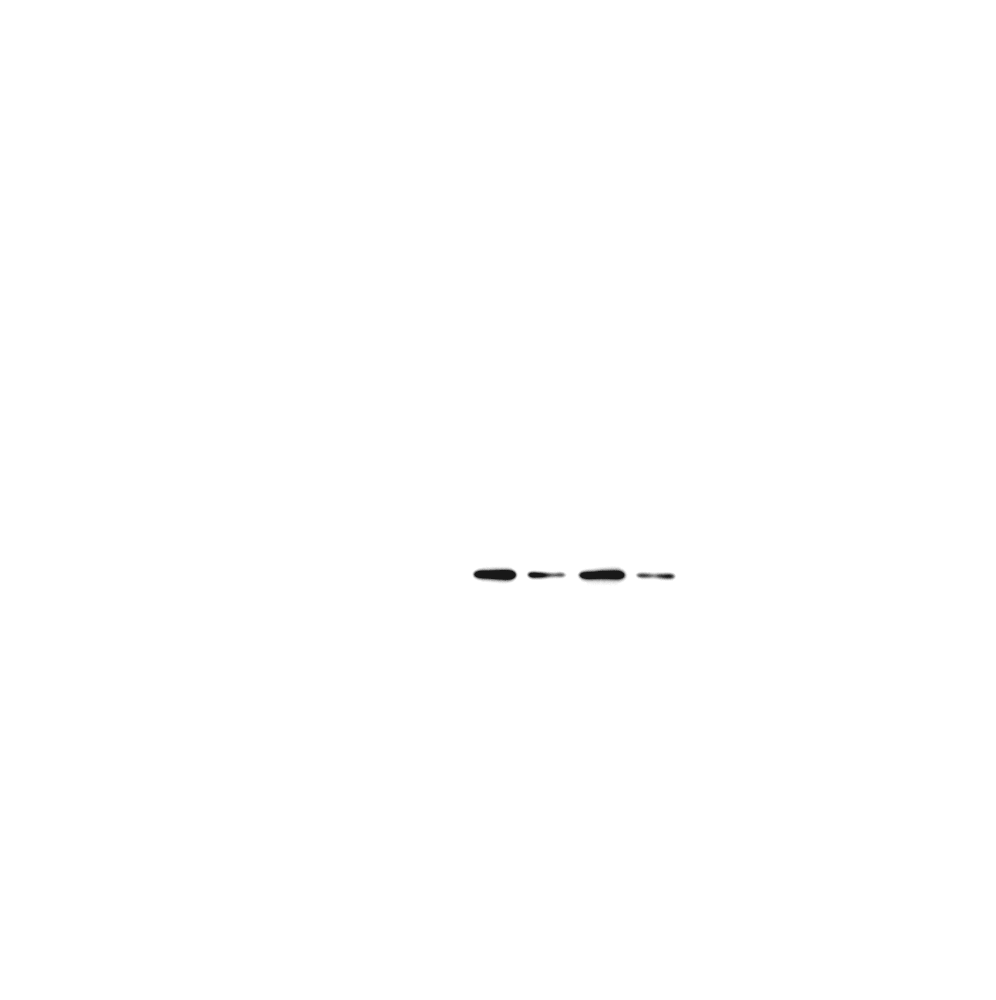 | | | |
| GAPDH | 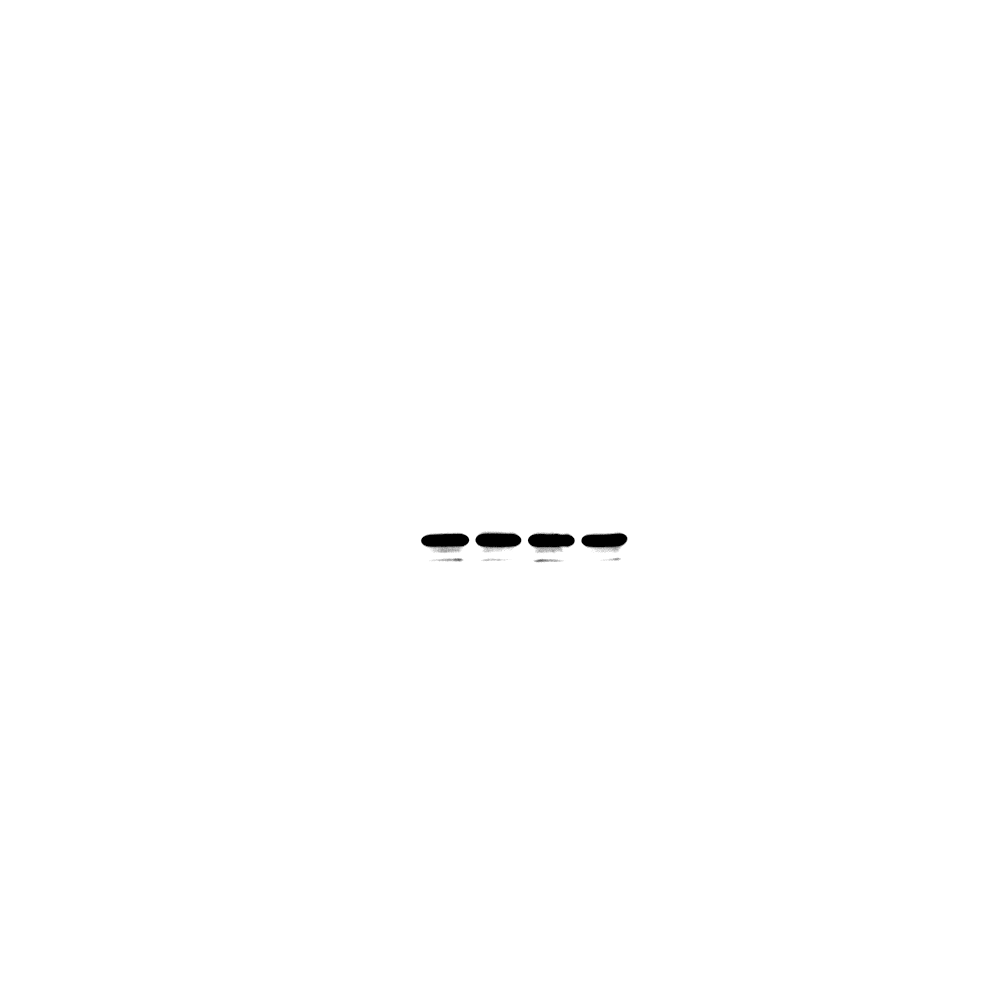 | | | |

**Table S5.** Figure 4H presents representative Western blots probing for PI3K, p-PI3K p85, AKT, p-AKT, and the corresponding loading control GAPDH in HTR2B-overexpressing HCAECs treated with MK2206.

|  | Control | MK2206 | MK2206+OE-HTR2B | MK2206+OE-NC |
| --- | --- | --- | --- | --- |
| PI3K | 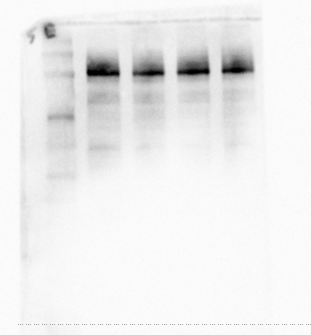 | | | |
| GAPDH | 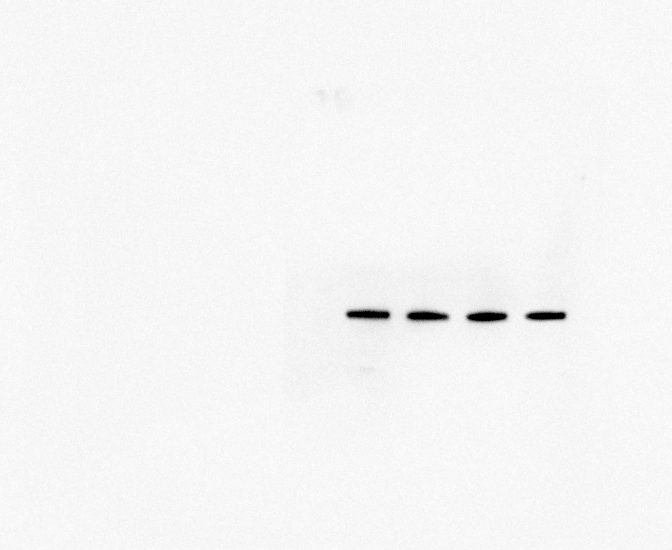 | | | |
| p-PI3K p85 | 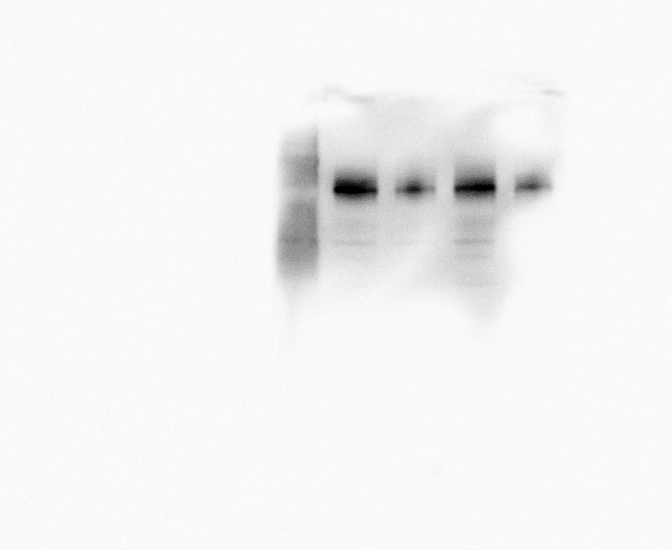 | | | |
| GAPDH | 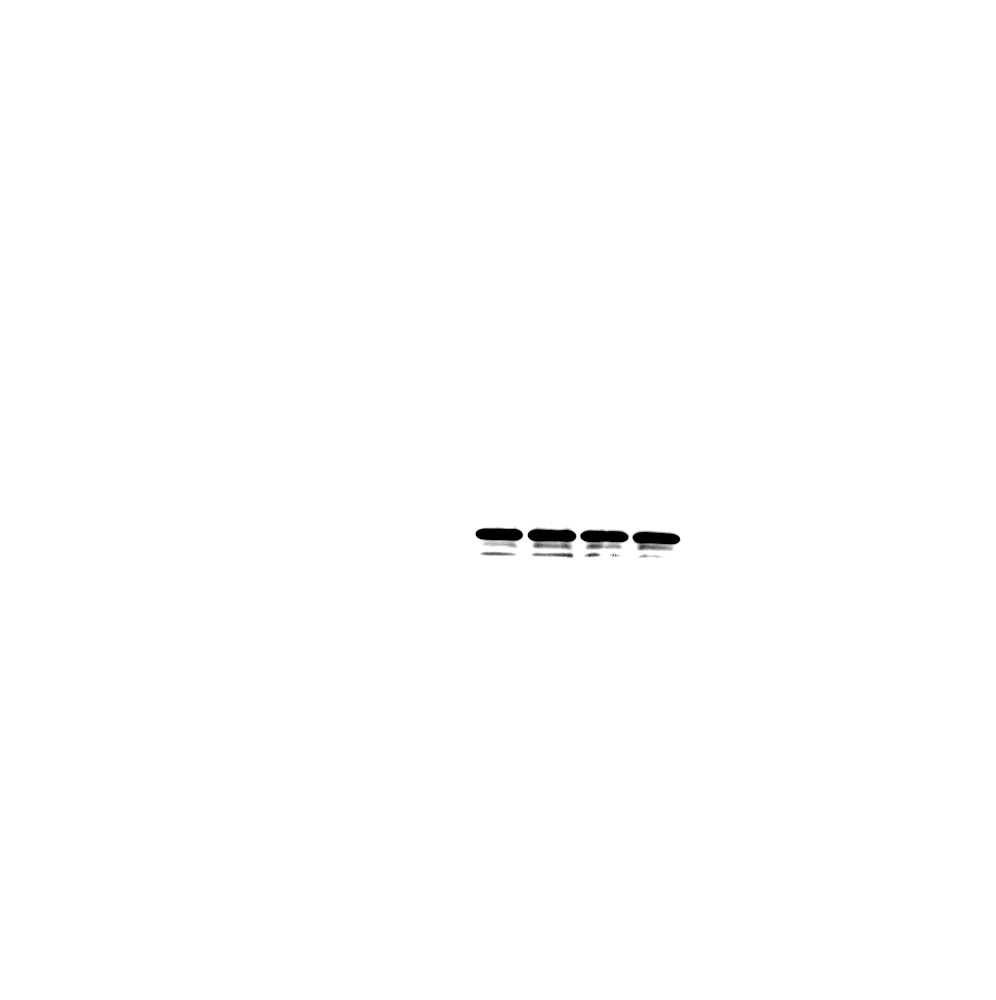 | | | |
| AKT | 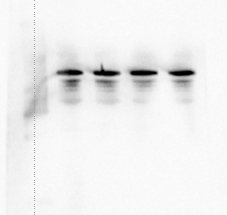 | | | |
| GAPDH | 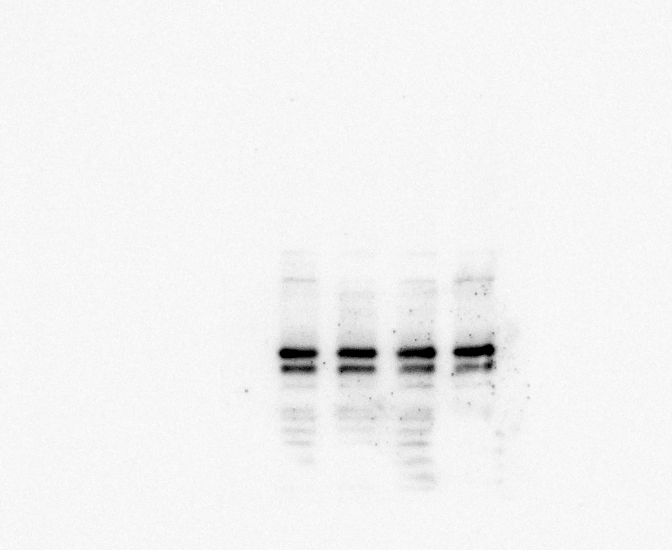 | | | |
| p-AKT | 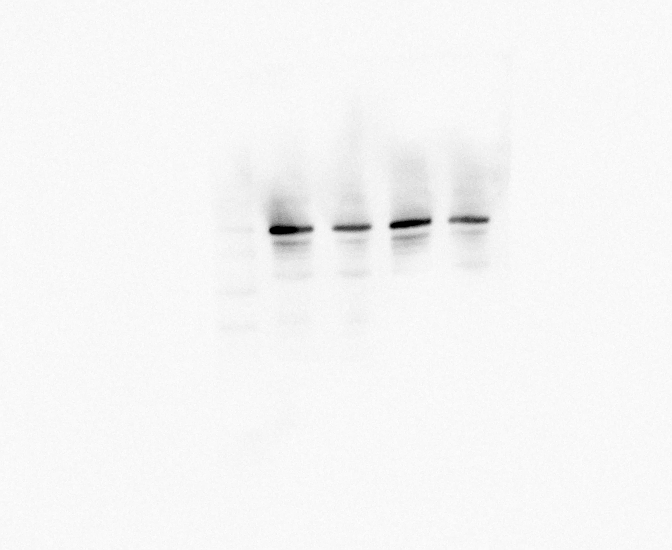 | | | |
| GAPDH | 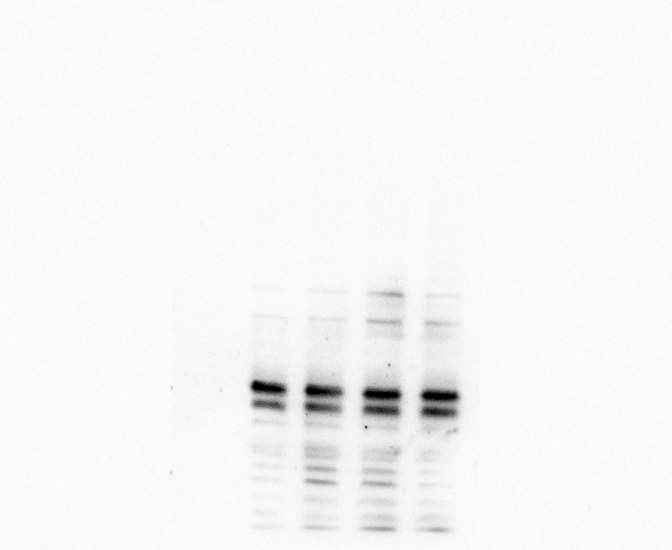 | | | |

**Table S6.** Figure 5A displays representative Western blots of PI3K, p-PI3K p85, AKT, p-AKT, and the corresponding loading control GAPDH in HCAECs treated with OX-LDL, 740Y-P, or Erastin individually or in combination.

|  | Control | OX-LDL | 740 Y-P | OX-LDL+740 Y-P | Erastin | Erastin+740 Y-P |
| --- | --- | --- | --- | --- | --- | --- |
| PI3K | 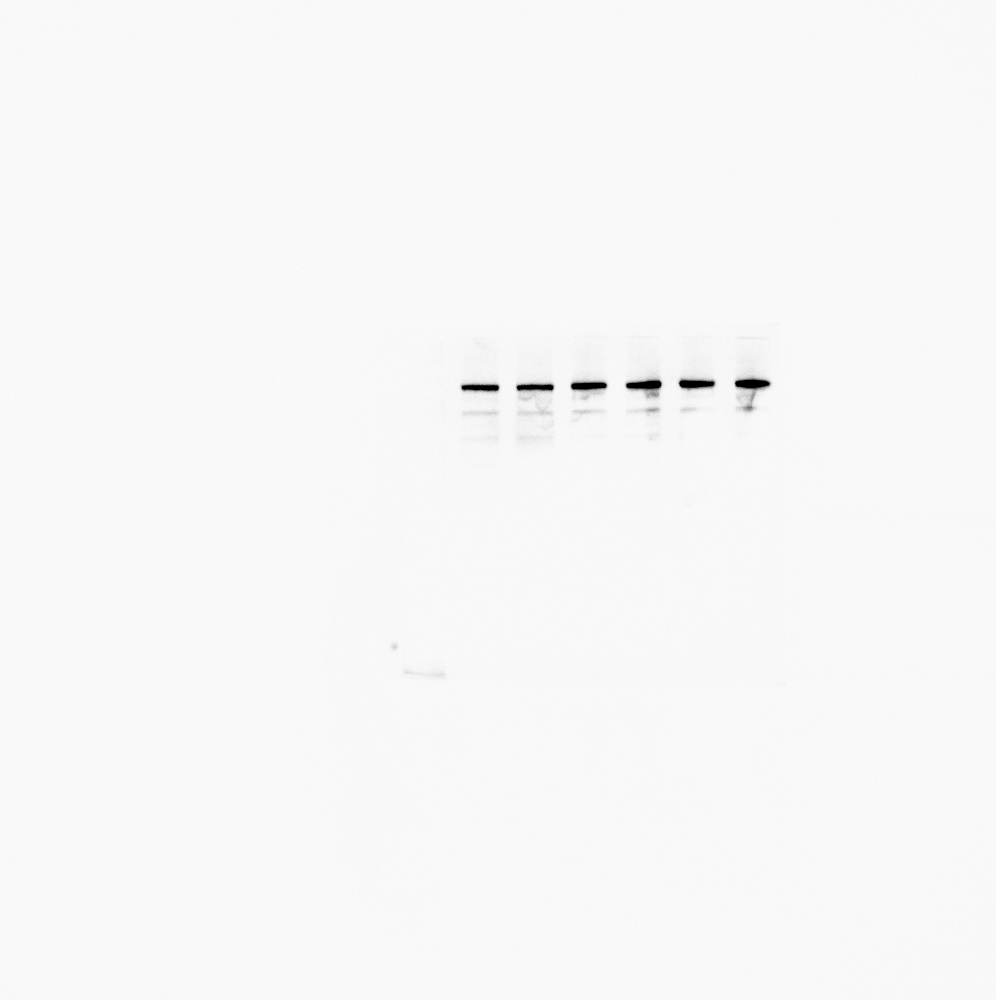 | | | | | |
| GAPDH | 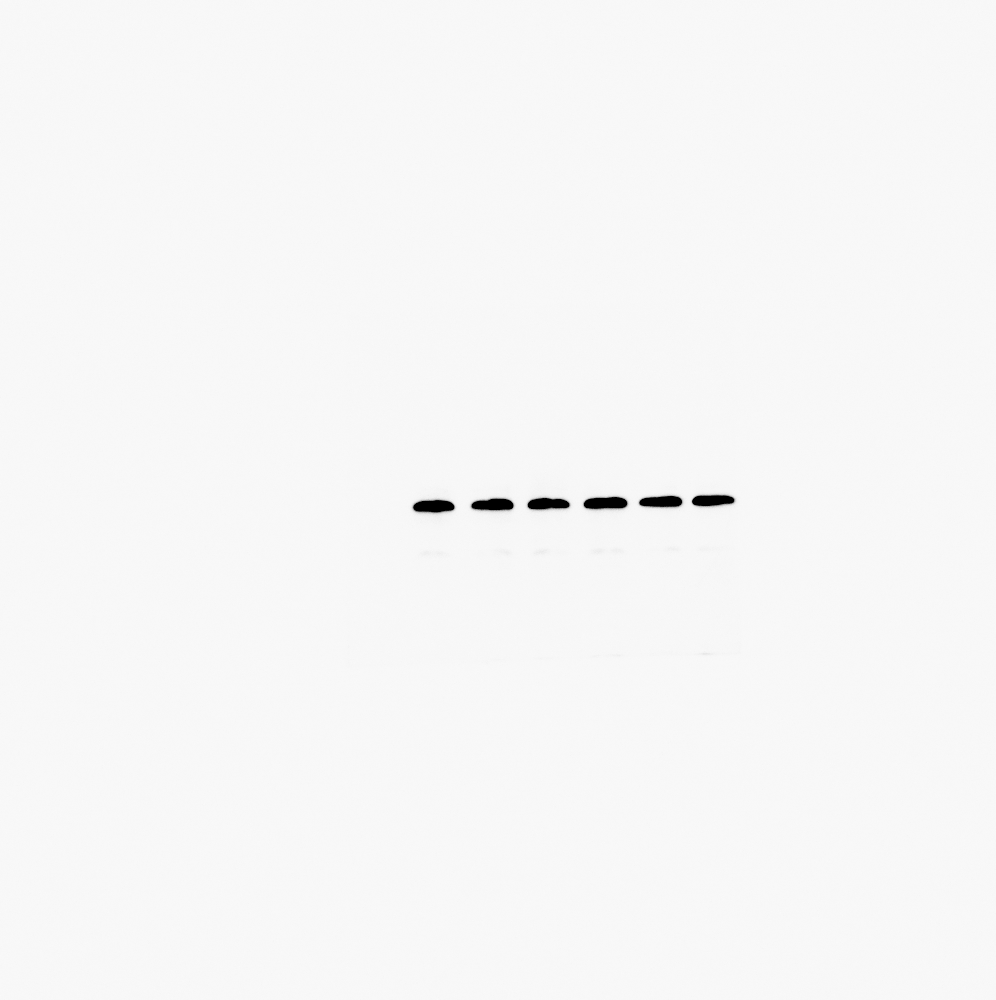 | | | | | |
| p-PI3K p85 | 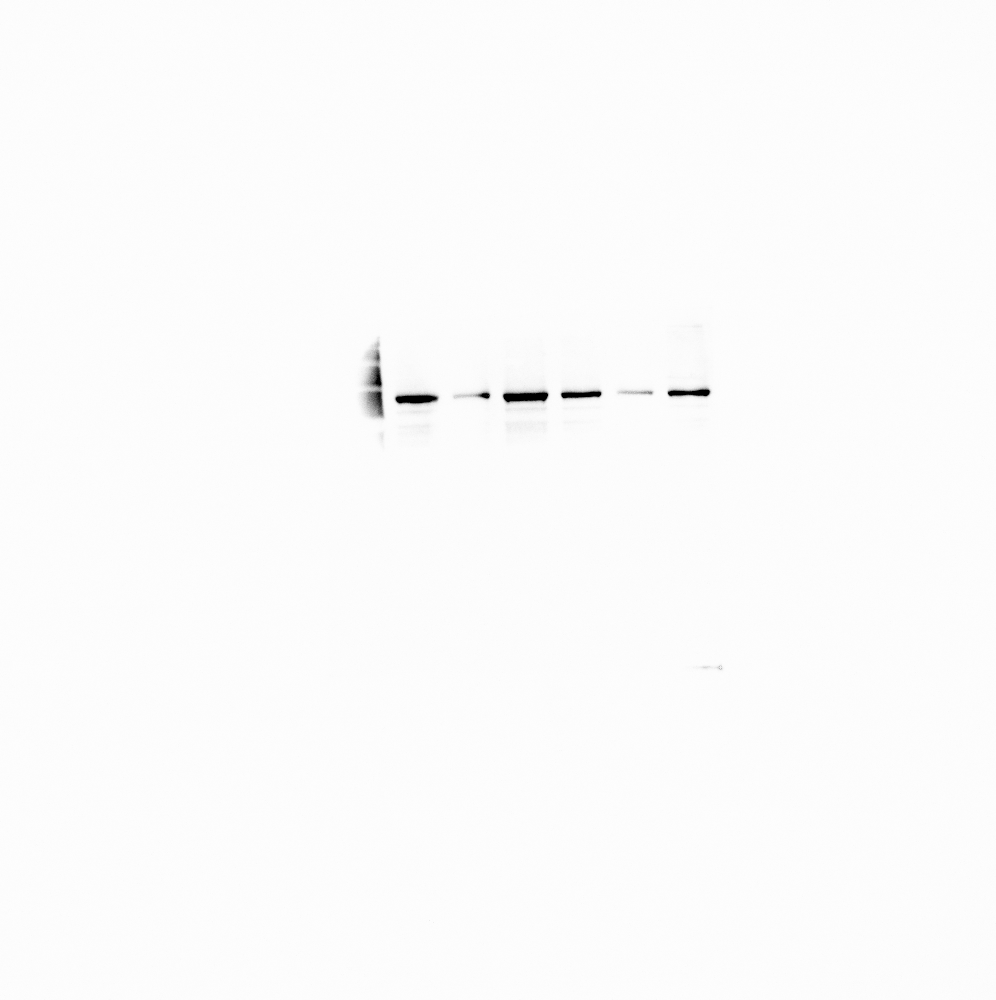 | | | | | |
| GAPDH | 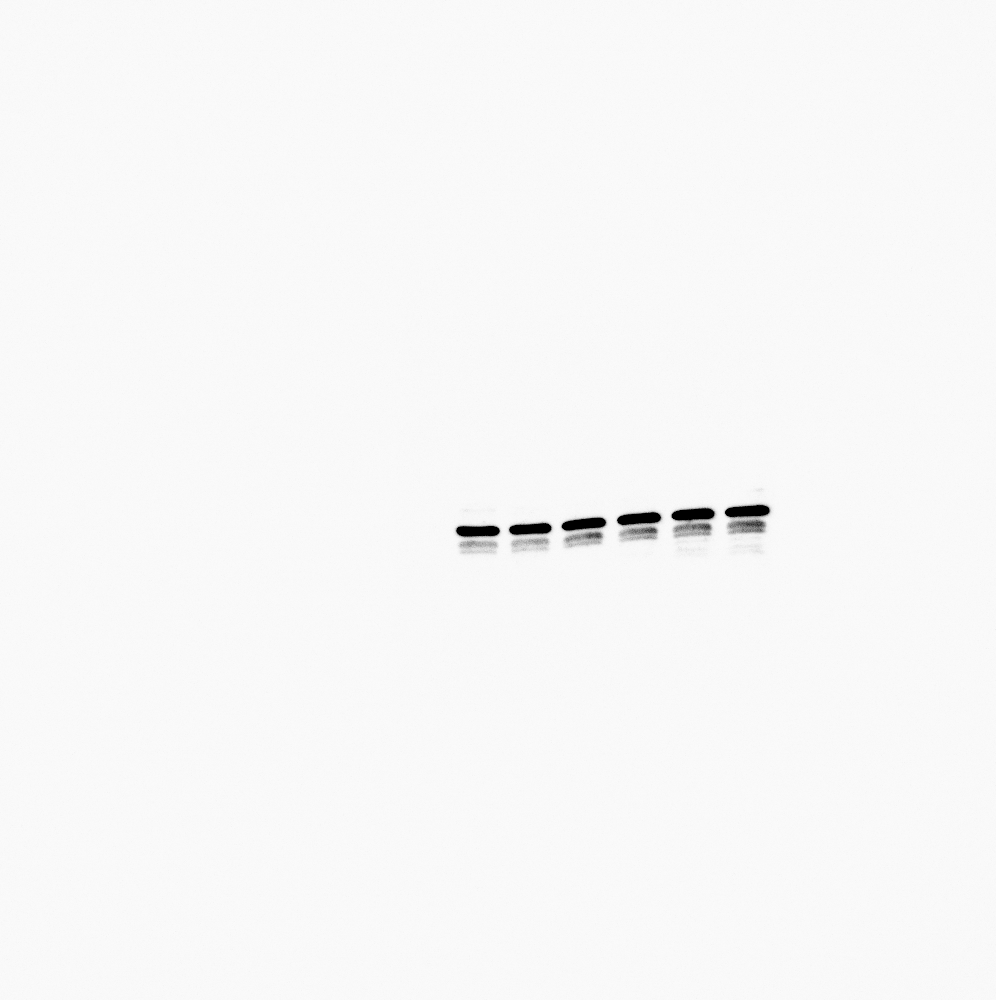 | | | | | |
| AKT | 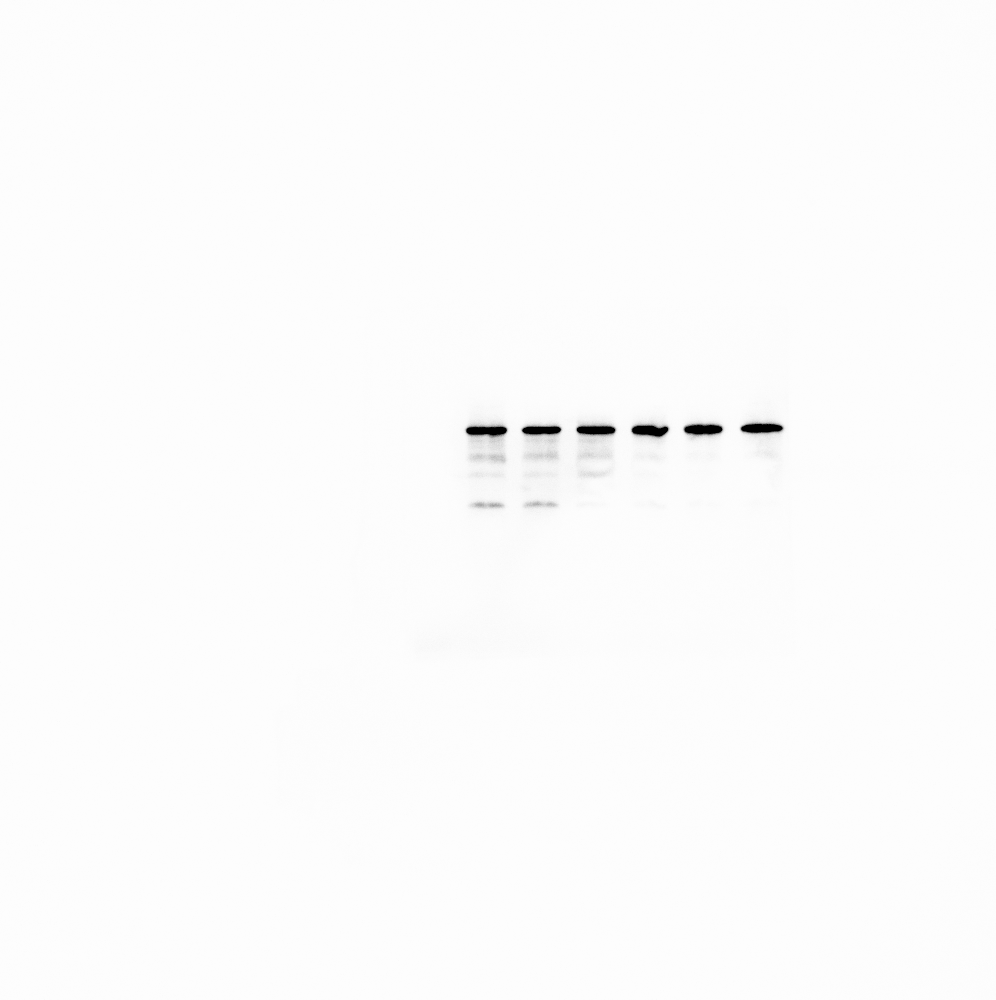 | | | | | |
| GAPDH | 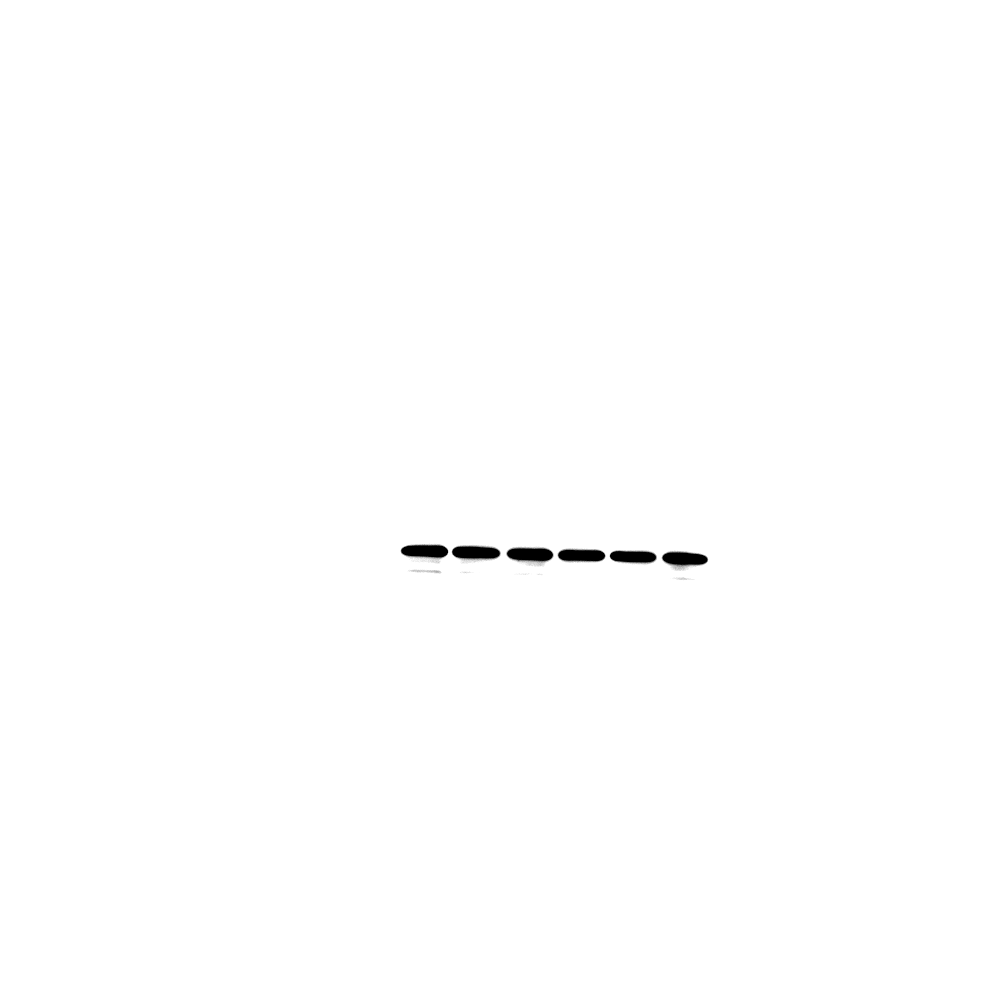 | | | | | |
| p-AKT | 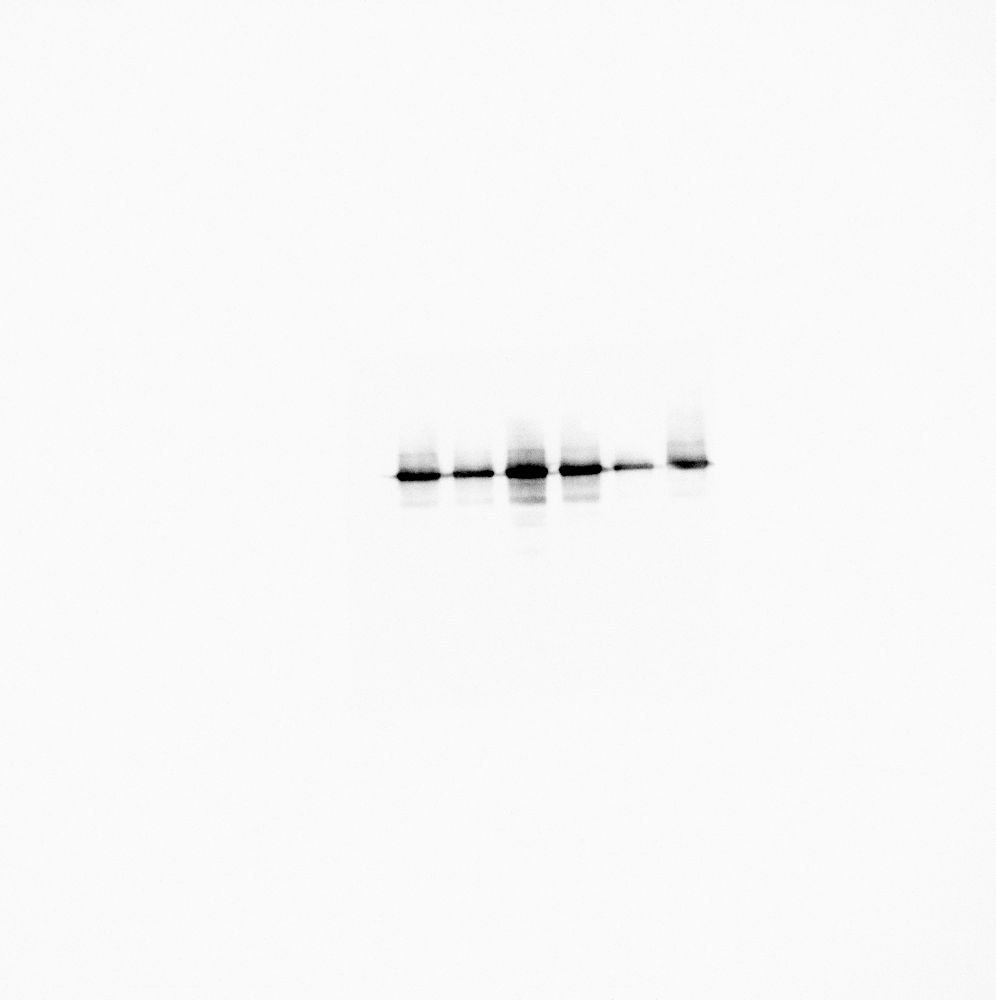 | | | | | |
| GAPDH | 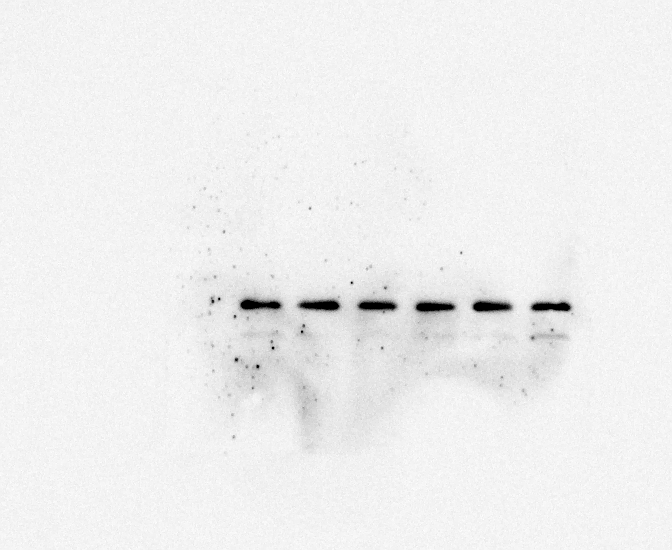 | | | | | |

**Table S7.** Figure 5I displays representative Western blots probing for GPX4 and SLC7A11, along with their corresponding loading control GAPDH, in HCAECs treated with OX-LDL, 740Y-P, or Erastin individually or in combination.

|  | Control | OX-LDL | 740 Y-P | OX-LDL+740 Y-P | Erastin | Erastin+740 Y-P |
| --- | --- | --- | --- | --- | --- | --- |
| GPX4 | 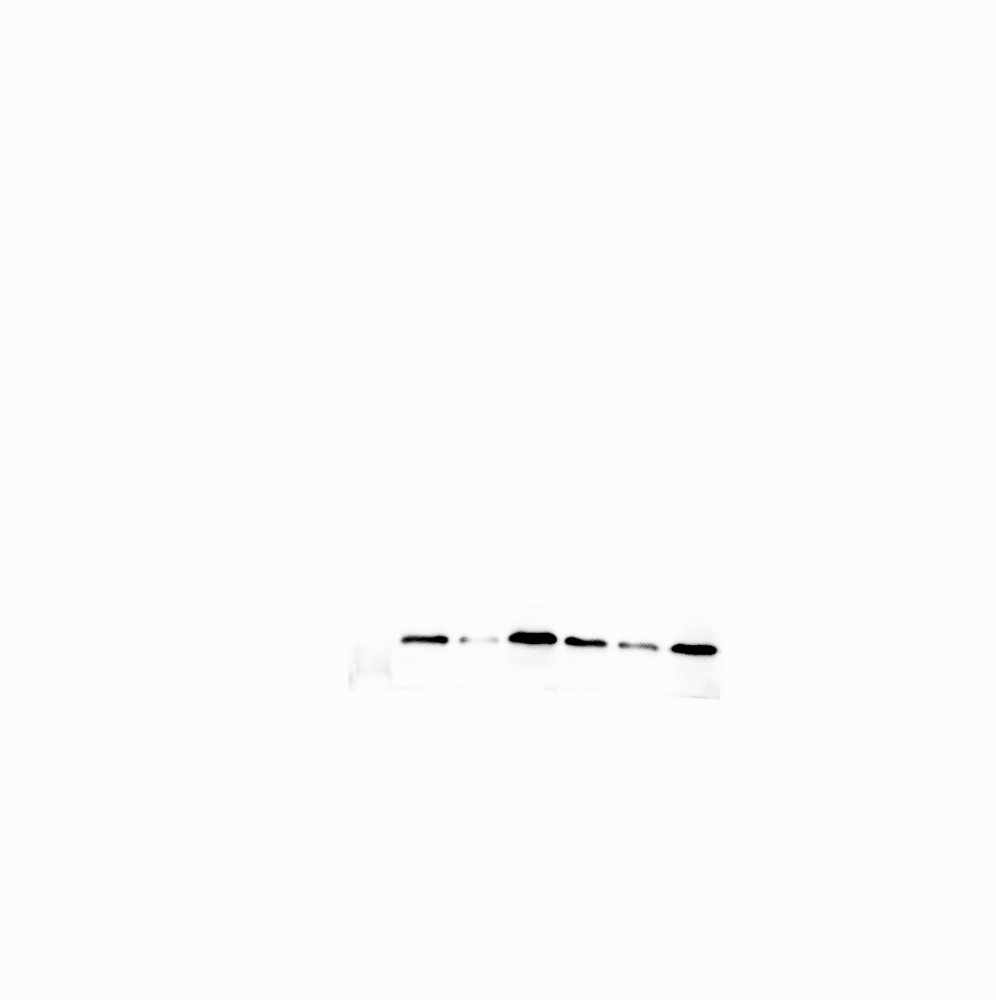 | | | | | |
| GAPDH | 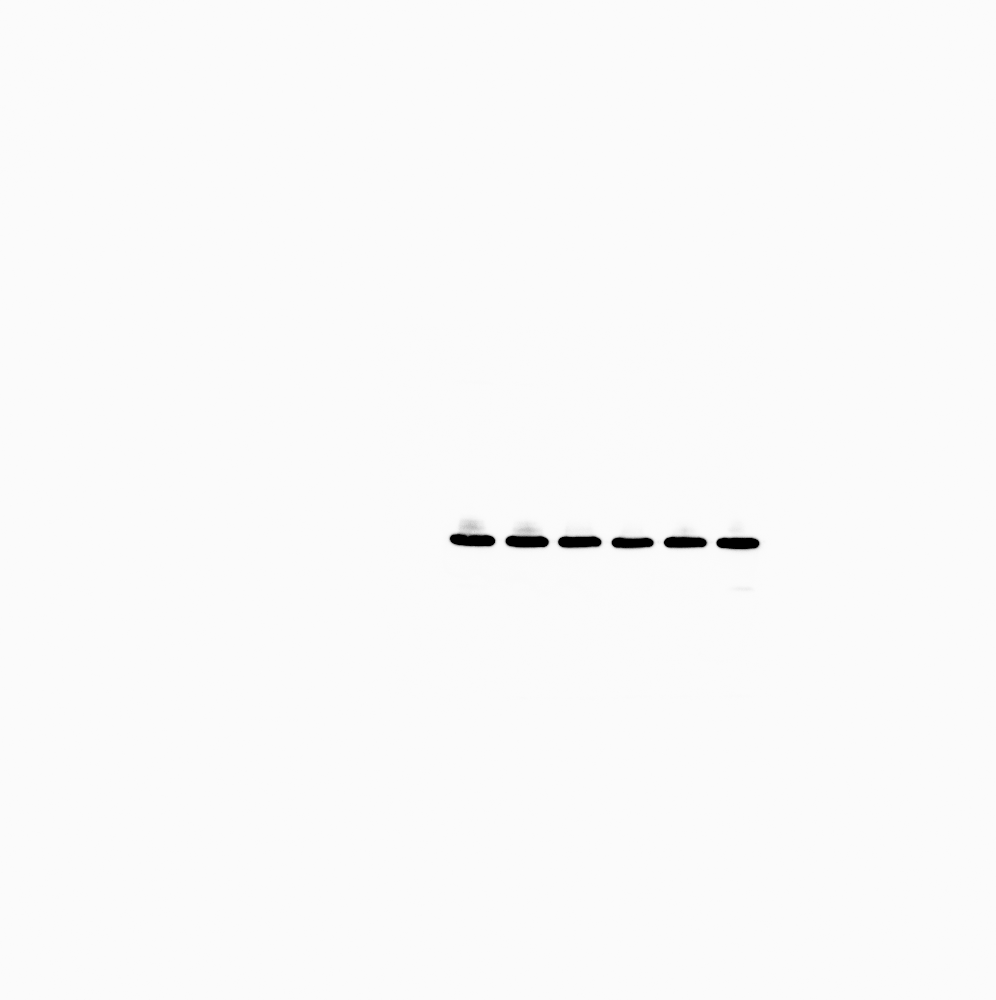 | | | | | |
| SLC7A11 | 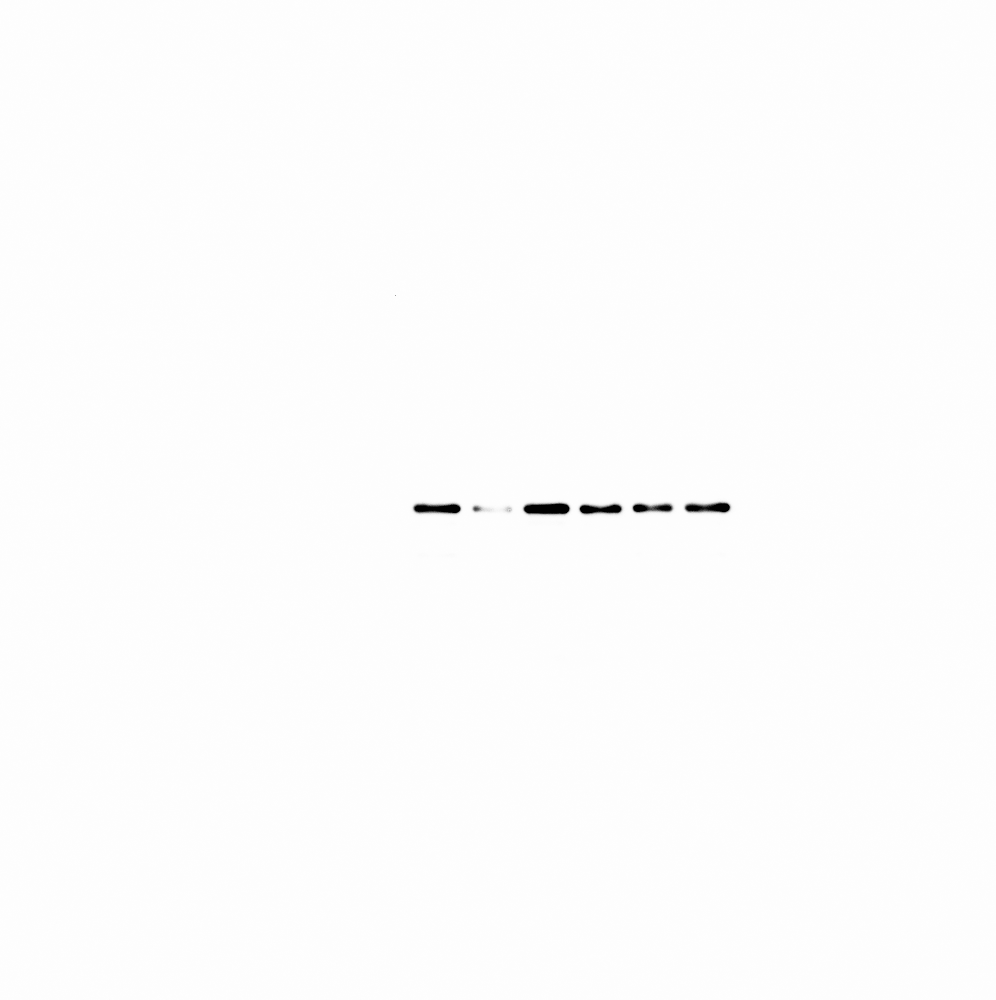 | | | | | |
| GAPDH | 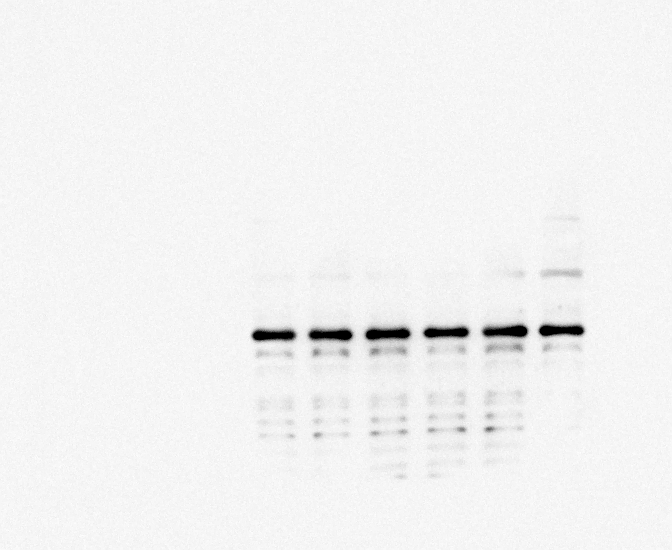 | | | | | |

**Table S8.** Figure 6F presents representative Western blots detecting GPX4, SLC7A11, PI3K, p-PI3K p85, AKT, p-AKT, and the corresponding loading control GAPDH in aortic tissues of ApoE^-/-^ mice treated with either OX-LDL alone or OX-LDL combined with BW-723C86.

|  | Control | OX-LDL | OX-LDL+BW-723C86 |
| --- | --- | --- | --- |
| GPX4 | 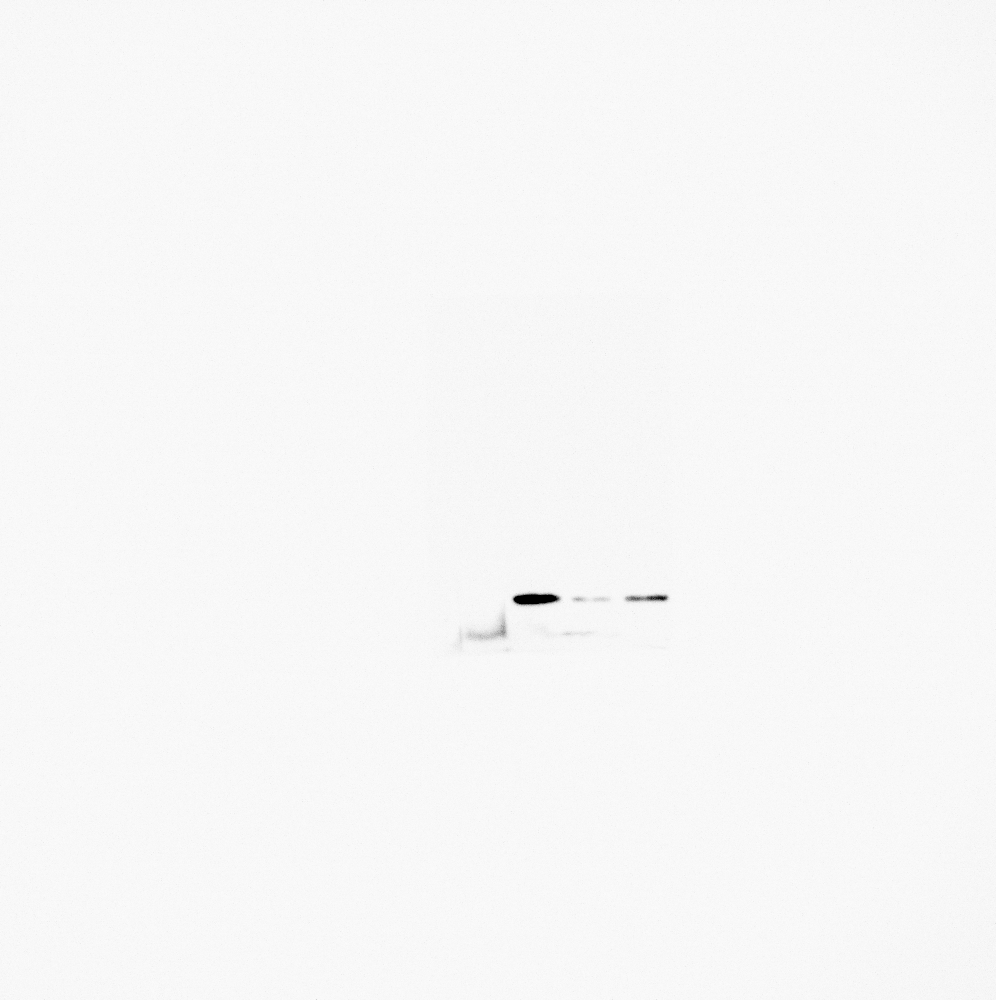 | | |
| GAPDH | 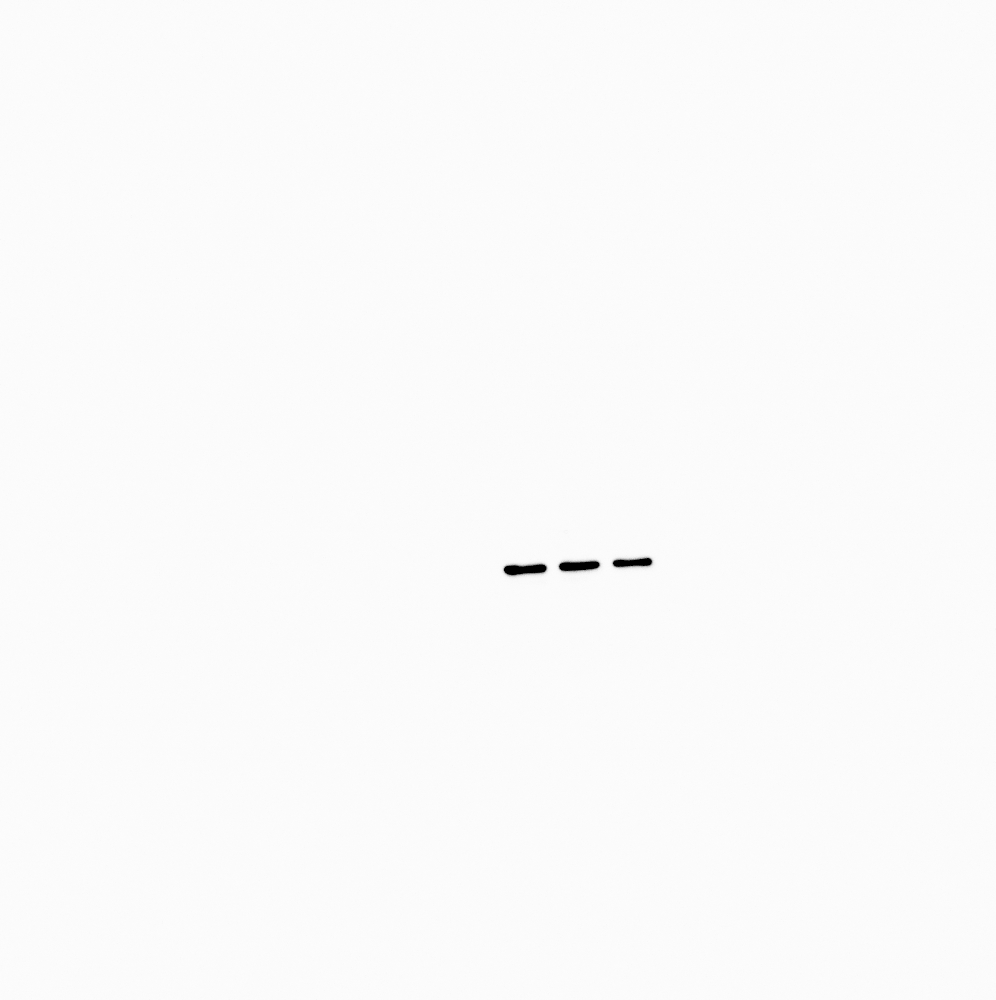 | | |
| SLC7A11 | 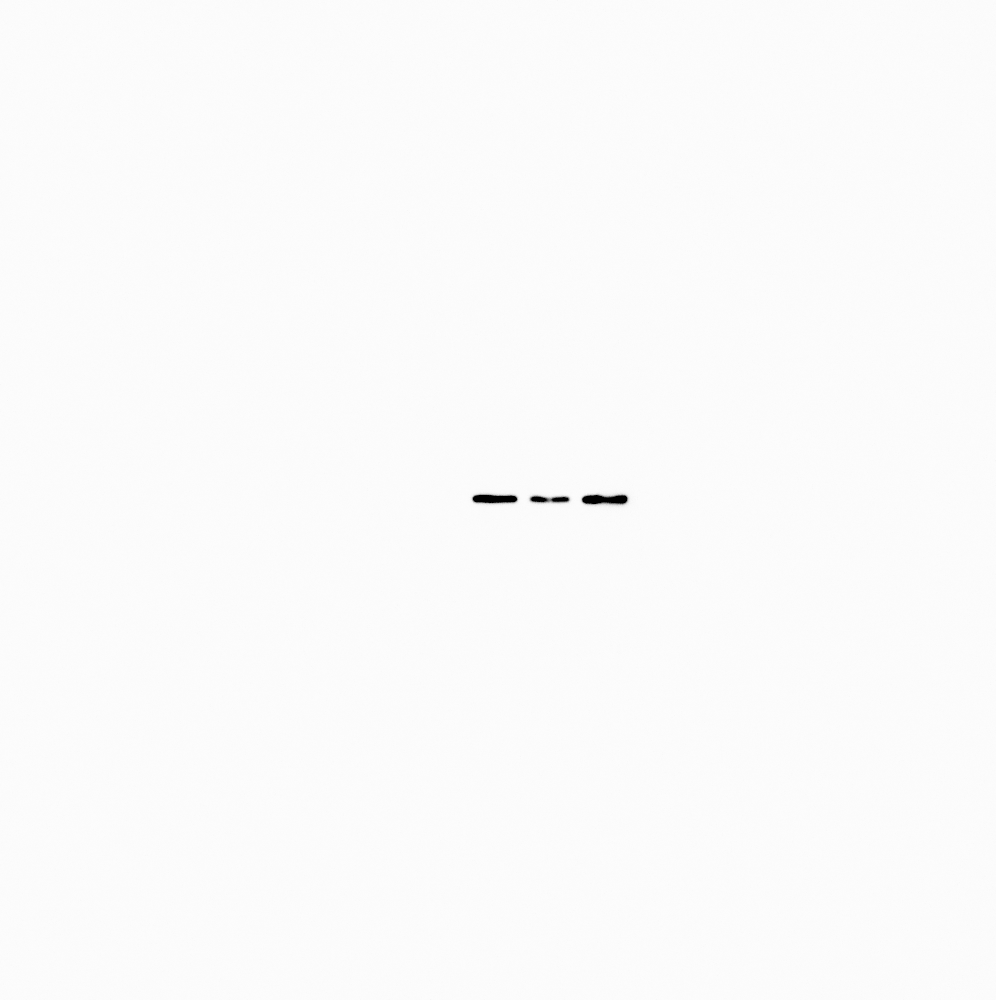 | | |
| GAPDH | 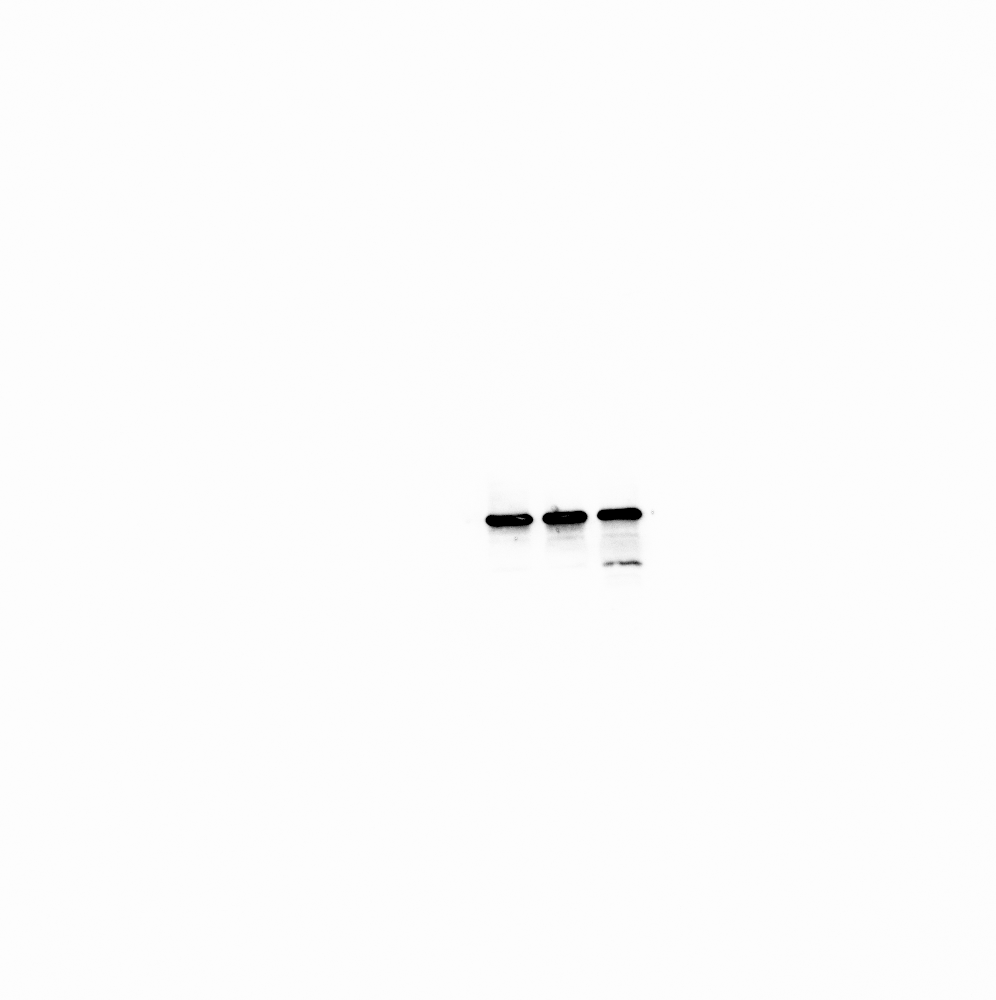 | | |
| PI3K | 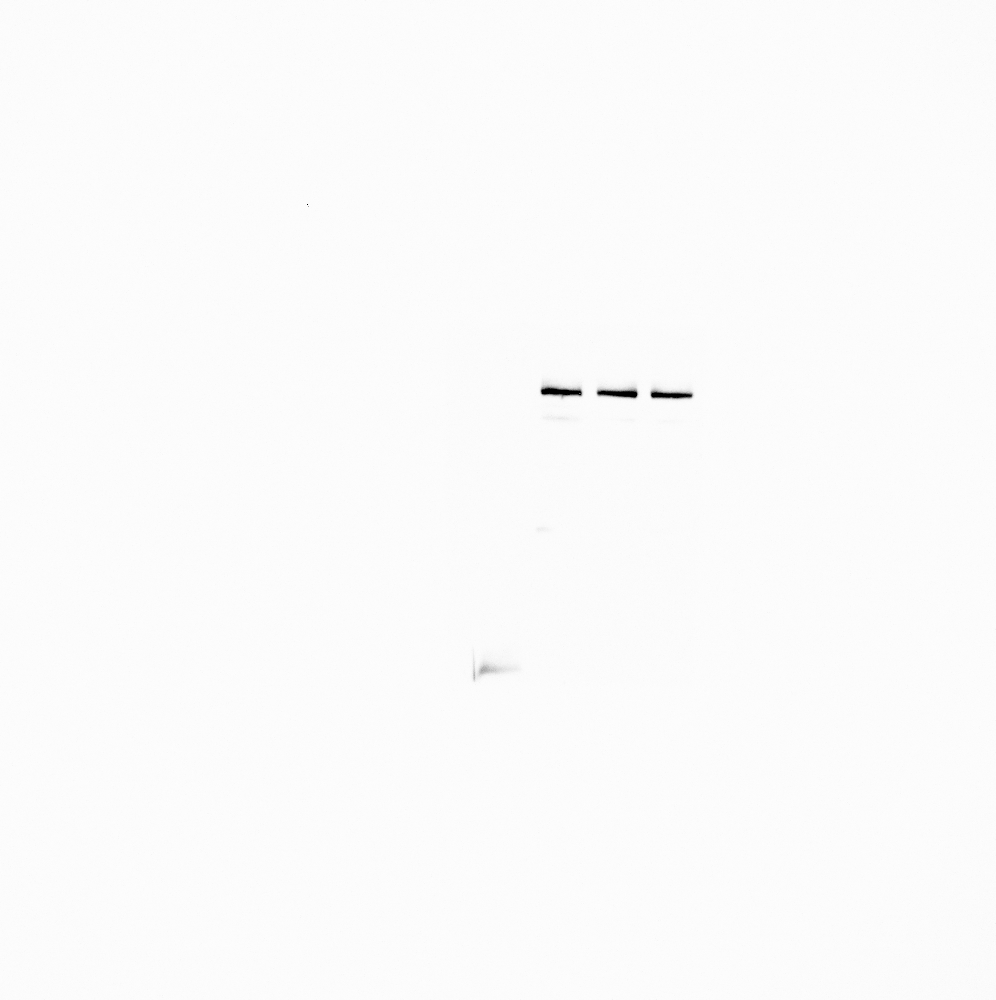 | | |
| GAPDH | 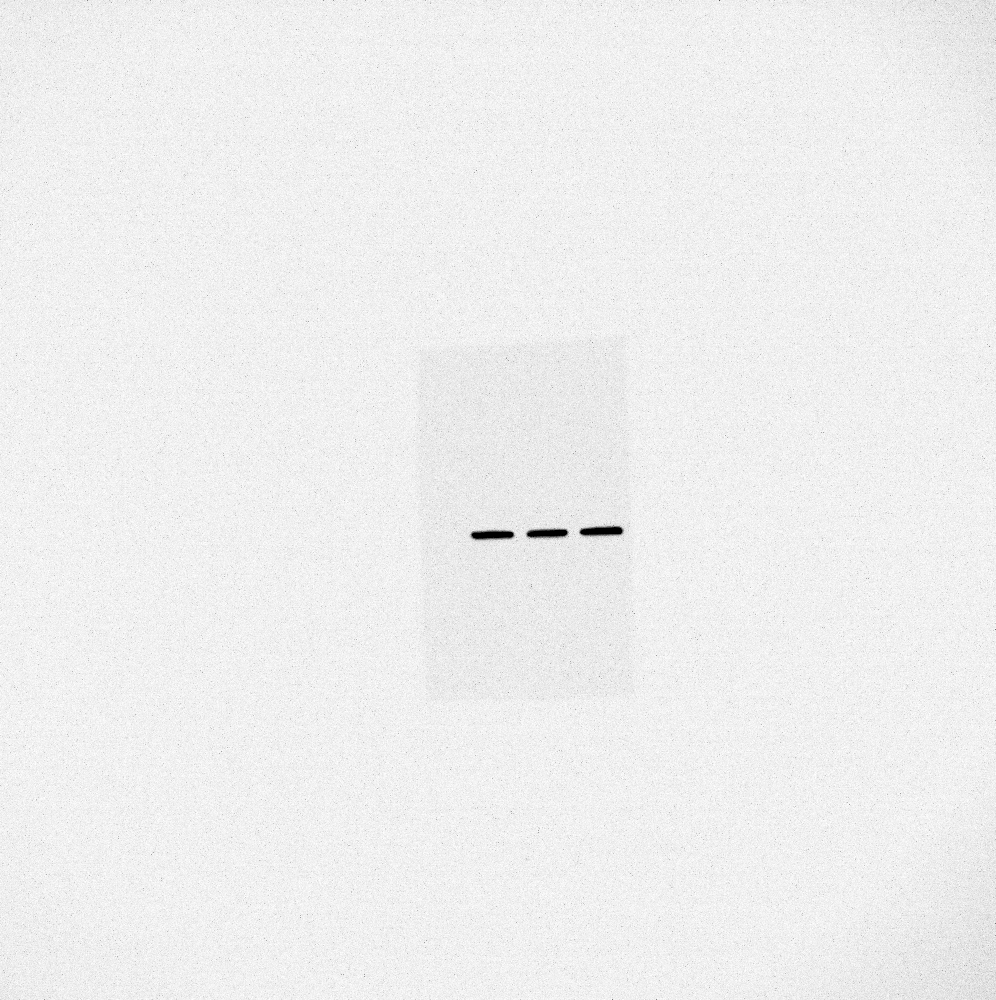 | | |
| p-PI3K p85 | 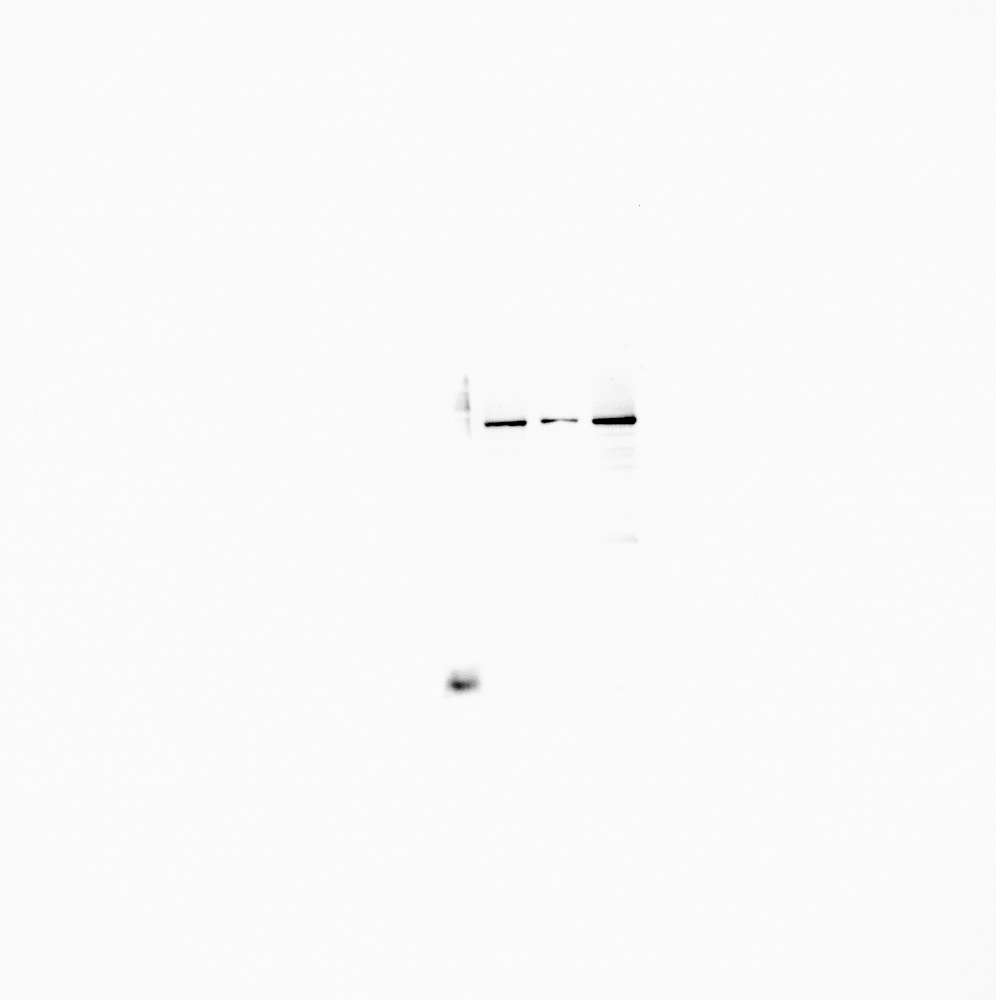 | | |
| GAPDH | 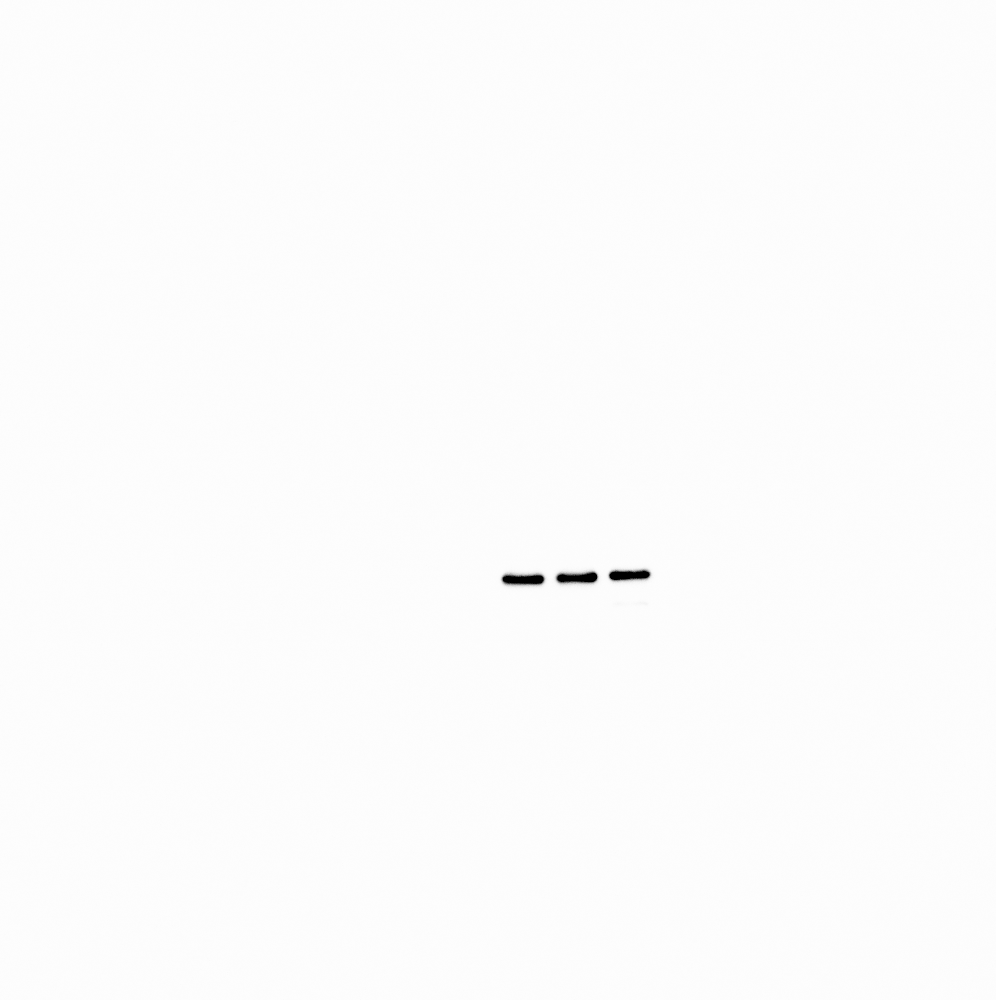 | | |
| AKT | 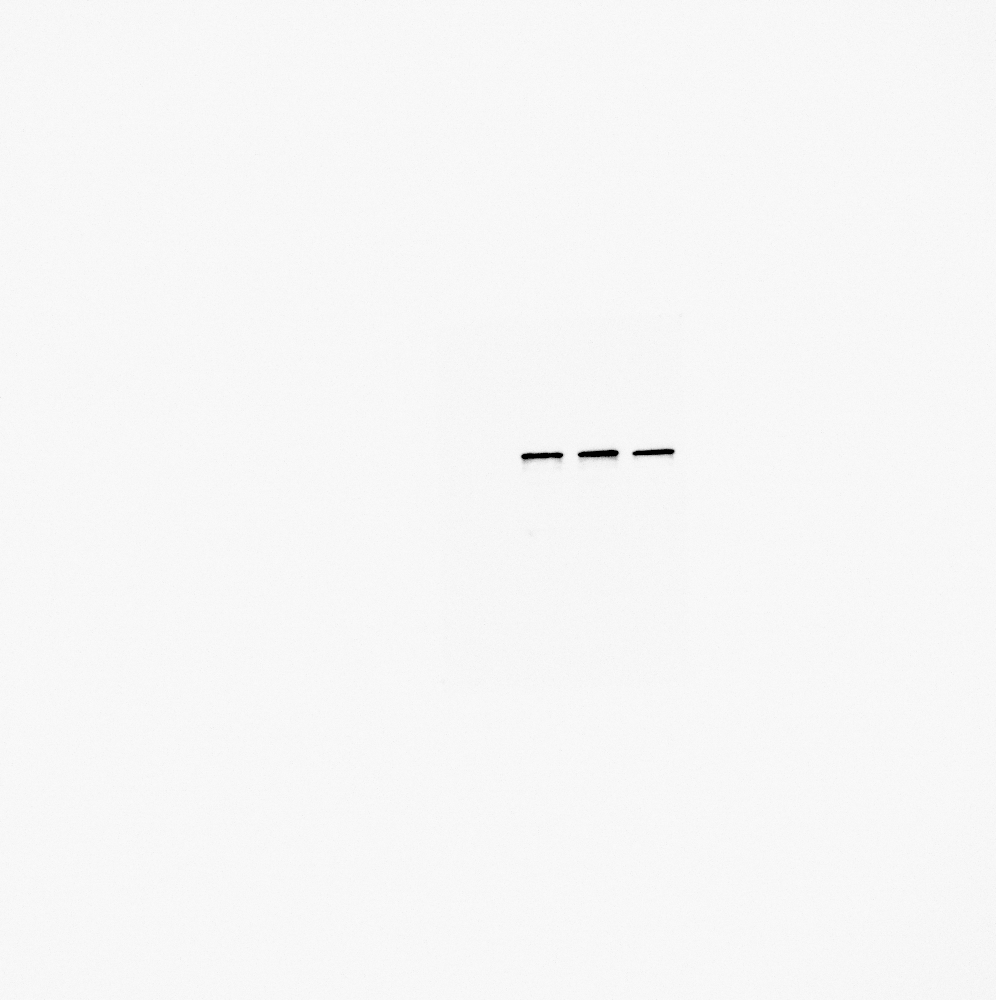 | | |
| GAPDH | 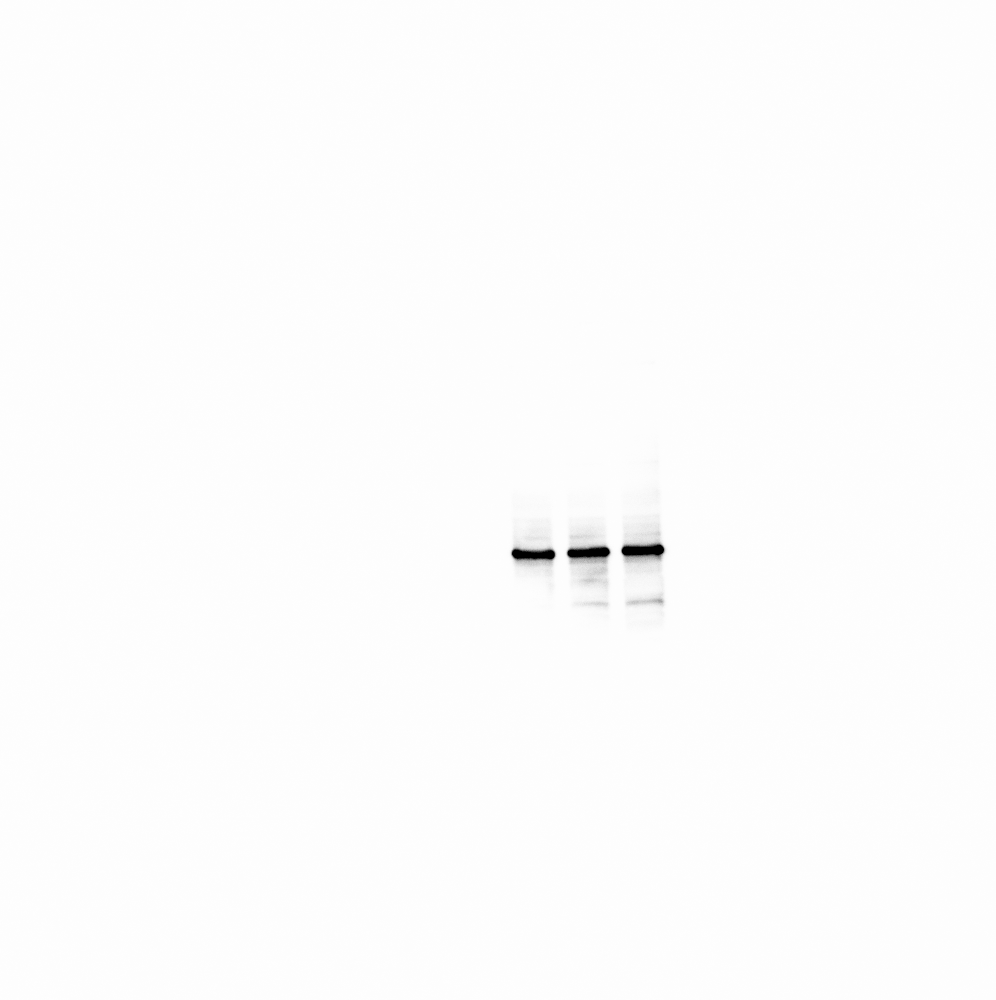 | | |
| p-AKT | 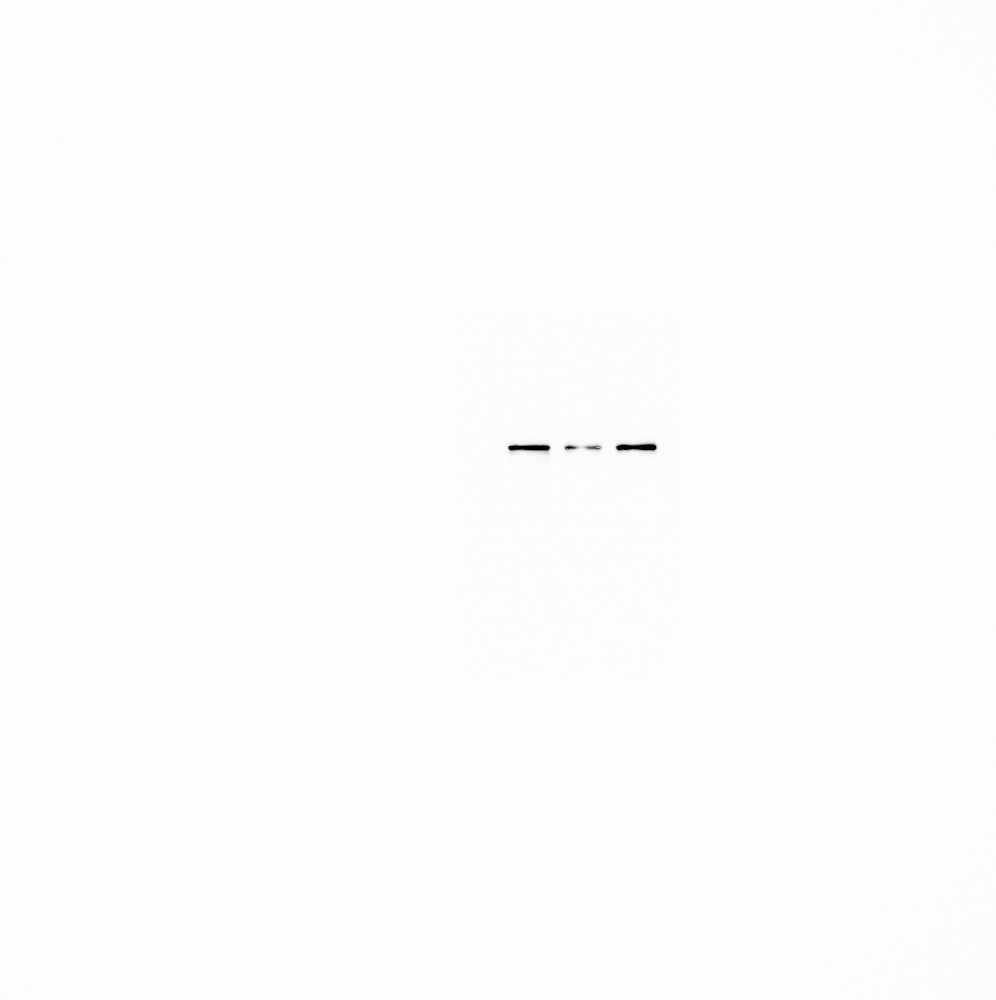 | | |
| GAPDH | 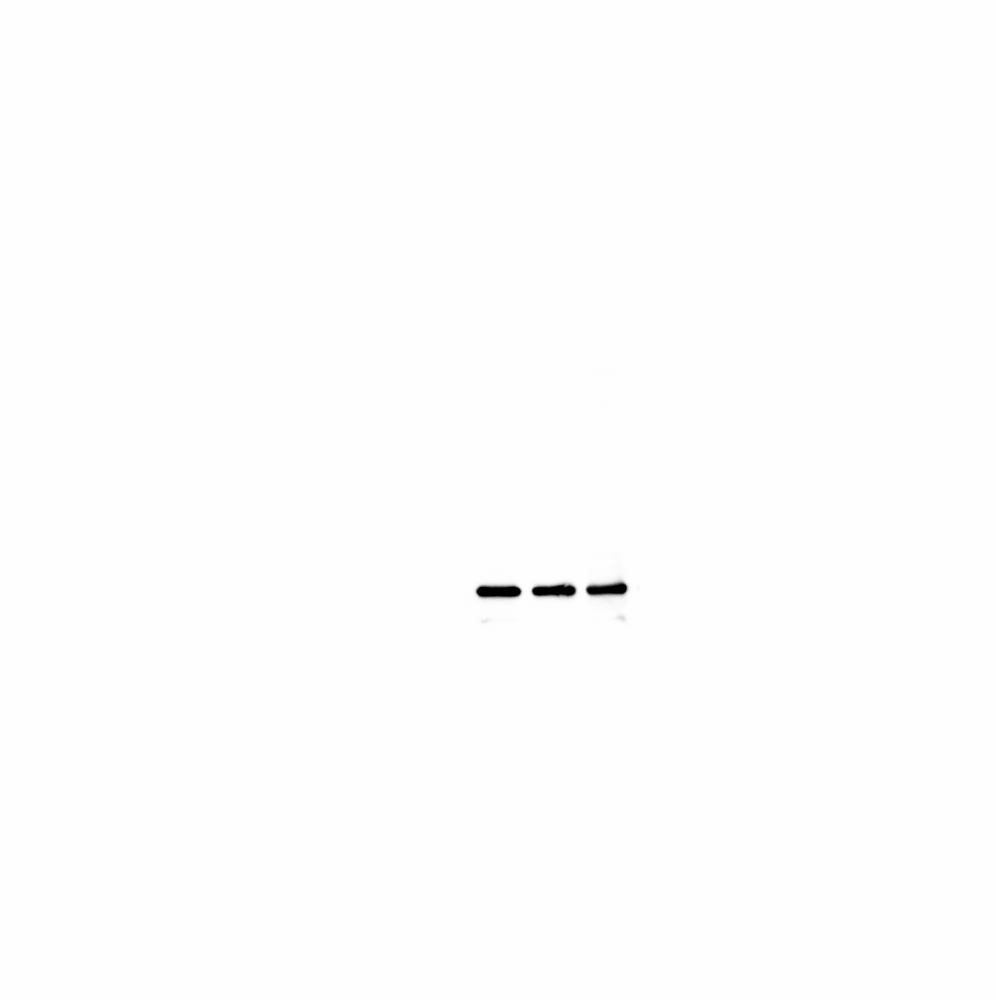 | | |
